# Heat stress promotes ferroptosis in Jiaji duck myocardium by disrupting iron homeostasis and inducing lipid peroxidation

**DOI:** 10.1016/j.psj.2026.107241

**Published:** 2026-06-09

**Authors:** Qijun Liang, Xinli Zheng, Tieshan Xu, Piaoran Liu, Yiyong Chen, Zhemin Lin, Lihong Gu

**Affiliations:** a Institute of Animal Science and Veterinary Medicine, Hainan Academy of Agricultural Sciences, Haikou 571100, China; b Tropical Crops Genetic Resources Institute, Chinese Academy of Tropical Agricultural Sciences, Haikou 571100, China; c Hainan Chuanwei Wenchang Chicken Industry Co., Ltd., Wenchang 571300, China

**Keywords:** Heat stress, Jiaji duck, Myocardial injury, Ferroptosis

## Abstract

Heat stress is a common environmental challenge in poultry production, causing substantial economic losses and acute mortality. The heart is highly sensitive to thermal load and is a major target of heat-induced injury. Although heat stress–related myocardial injury in poultry has been linked to oxidative stress and apoptosis, the contribution of ferroptosis remains unclear. Here, we used the Jiaji duck as a model to investigate the molecular mechanisms of heat stress–induced myocardial injury. We established an acute heat stress model in Jiaji ducks (40 ± 1°C, 80% relative humidity, 3 h) and an in vitro heat stress model in primary cardiomyocytes, and performed a multi-platform analysis. Acute heat stress reduced survival in a time-dependent manner, increased serum CK-MB and LDH levels, and induced myocardial lesions characterized by myofiber disorganization and disruption. Transmission electron microscopy revealed mitochondrial shrinkage and loss of cristae. Whole-proteome profiling identified 1,011 differentially expressed proteins, with enrichment of ferroptosis-related terms and pathways and concurrent enrichment of AMPK signaling. Biochemical analyses showed increased ROS and MDA levels and elevated ferrous iron (Fe²⁺), accompanied by glutathione (GSH) depletion and reduced GPX4 abundance. Consistently, western blotting indicated activation of the AMPK/ULK1 axis (increased p-AMPK and p-ULK1), upregulation of TFR1 and ACSL4, and downregulation of FTH1 and SLC7A11. In vitro, heat stress elicited similar molecular changes together with increased CK-MB/LDH release and reduced viability, supporting a direct cardiomyocyte response. Collectively, these findings indicate that heat stress promotes a ferroptosis-like injury program in Jiaji duck cardiomyocytes, characterized by iron accumulation, impaired GSH–GPX4 antioxidant defense, and enhanced lipid peroxidation, and is associated with activation of AMPK/ULK1 signaling. These results provide mechanistic insights and potential targets for mitigating heat stress–induced myocardial injury in poultry.

## Graphical abstract

**Figure:**
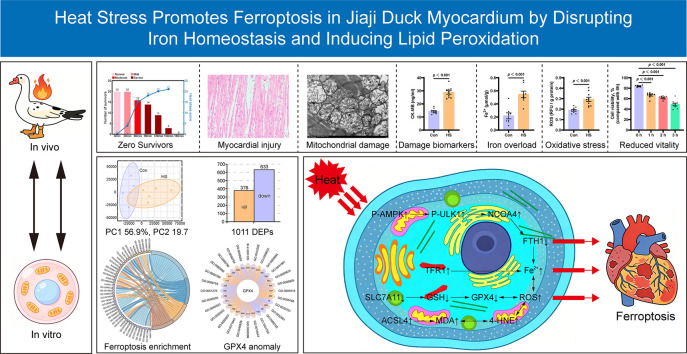

## Introduction

As poultry production becomes increasingly intensive and large-scale, and as summer heatwaves and humidity become more frequent under global warming, heat stress in poultry is becoming more prevalent and severe (Nawab et al., 2018; Oluwagbenga and Fraley, 2023). As homeothermic animals, poultry have a limited thermoneutral zone (TNZ). However, because they are covered in feathers and lack sweat glands, they dissipate heat primarily through evaporative cooling via panting and by regulating peripheral blood flow. Consequently, under hot and humid conditions, poultry are prone to reduced heat loss, elevated core body temperature, and increased metabolic burden (Oladokun and Adewole, 2022). When ambient temperature persistently exceeds the upper limit of the TNZ, physiological thermoregulation is rapidly overwhelmed and transitions to a stress response. This shift disrupts redox homeostasis and electrolyte balance and can cause pathological injury in multiple organs, ultimately reducing productivity and, in severe cases, resulting in sudden death (Madkour et al., 2022). Among these organs, the heart is particularly vulnerable to heat stress because of its high metabolic demand. Cardiac injury is often accompanied by structural damage and functional impairment, directly threatening poultry survival (Xu et al., 2019; Yao et al., 2025).

Myocardial tissue is highly sensitive to thermal stress because sustained, high-intensity contraction requires a stable energy supply and tightly controlled redox homeostasis (Bertero and Maack, 2018). In cardiomyocytes, ATP is generated primarily through mitochondrial oxidative phosphorylation. Heat exposure accelerates metabolism and oxygen consumption and can simultaneously impair mitochondrial structure and function, thereby reducing electron transport efficiency and increasing reactive oxygen species (ROS) production (Li et al., 2023a). When ROS generation exceeds cellular antioxidant capacity, oxidative damage to lipids and proteins accumulates rapidly. This process compromises membrane integrity and ion homeostasis, ultimately leading to cardiomyocyte dysfunction and necrotic-like injury (Tsutsui et al., 2011). Although previous studies have shown that heat stress induces oxidative stress and metabolic dysregulation in the myocardium, the dominant cell-death pathways and their regulatory networks underlying heat stress–induced myocardial injury in poultry remain poorly defined. In particular, mechanisms involving iron metabolism and membrane lipid peroxidation have received limited attention.

During heat stress, cells typically activate a range of protective responses. A central component of this defense is the upregulation of heat shock proteins (HSPs), which helps maintain proteostasis by facilitating the refolding or clearance of misfolded proteins and preventing aberrant aggregation (Gouda et al., 2024; Jacob et al., 2017). However, accumulating evidence suggests that when stress is severe or prolonged, adaptive programs may be insufficient and cells can instead engage regulated cell-death pathways (Gonzalez-Rivas et al., 2020; Richter et al., 2010). Ferroptosis is a regulated form of cell death driven by iron-dependent lipid peroxidation. It is characterized by increased intracellular labile iron, excessive reactive oxygen species (ROS) generation and lipid peroxidation, glutathione (GSH) depletion, and compromised antioxidant defenses, particularly glutathione peroxidase 4 (GPX4) (Li et al., 2024). In mammalian cardiovascular disorders, including ischemia–reperfusion injury and heart failure, ferroptosis has been implicated as a contributor to myocardial damage (Wu et al., 2021; Zhao et al., 2025). In poultry, especially under heat stress, it remains unresolved whether ferroptosis contributes to myocardial injury and how energy stress intersects with dysregulated iron homeostasis.

Jiaji ducks are an important tropical meat-duck breed in China and have long adapted to hot and humid climatic conditions. Accordingly, they provide a suitable model for investigating avian heat adaptation and the mechanisms of heat stress–induced injury (Gu et al., 2020; Zhang et al., 2025). To date, studies of heat stress–related myocardial injury in poultry have focused largely on oxidative stress and apoptosis. However, systematic evidence is still limited regarding whether ferroptosis acts as a central effector of this injury and how upstream signaling pathways and iron-metabolism regulatory networks jointly drive myocardial damage. To address these gaps, we established an acute heat-stress model in Jiaji ducks in vivo and complemented it with an in vitro heat-stress system using primary cardiomyocytes. We then integrated histopathology, ultrastructural assessment, proteomics with bioinformatic enrichment analyses, ferroptosis-related biochemical measurements, and validation of key proteins to delineate myocardial pathological responses to thermal load.

## Materials and methods

### Animal treatment and sample collection

This study included 40 sixty-day-old Jiaji ducks purchased from the Chuanwei Jiaji Duck Breeding Base (Qionghai, China). All ducks were within the normal weight range for the batch and were maintained under standard conditions (22 ± 2°C; 60 ± 10% relative humidity; 12-h light/12-h dark cycle) with ad libitum access to feed and water. Healthy ducks with similar body weights were selected for the experiment and fasted for 12 h before treatment; drinking water remained available during this period. Ducks were randomly assigned to two groups (n = 20 per group): a control group (Con) without heat stress and a heat-stress group (HS). A heat-stress behavioral scoring sheet was developed based on previous chicken heat-stress experiments and published literature (Additional File 1) (Liang et al., 2024; Oladokun and Adewole, 2022; Shi et al., 2024). During heat exposure, clinical signs and behaviors were monitored continuously by a dedicated investigator, and behavioral scores were recorded every 30 min. Ducks in the HS group were housed in an intelligent artificial climate chamber at 40 ± 1°C and 80% relative humidity for up to 3 h; this relative humidity was chosen to approximate the hot and humid climatic conditions in which Jiaji ducks are commonly raised, rather than to introduce a separate humidity-deprivation stressor. Drinking water was not intentionally withdrawn during the heat-stress period. Control ducks were euthanized and sampled under thermoneutral conditions, whereas HS ducks were sampled according to individual terminal outcomes during heat exposure as described below.

For the Con group, blood samples were collected via the wing vein while ducks were alive, followed immediately by euthanasia and necropsy. For the HS group, each duck was monitored continuously throughout heat exposure. When a duck became moribund but was still alive, blood was collected immediately from the wing vein; after death occurred, the carcass was removed from the chamber without delay and heart tissue was sampled within minutes. Thus, no HS duck remained in the 40°C environment after death, which minimized post-mortem autolysis and protein degradation. All blood samples used in this study were collected ante-mortem via the wing vein, and no visible hemolysis was observed during collection. Whole blood was allowed to clot at room temperature for 1 h and then centrifuged to obtain serum, which was aliquoted and stored at −80°C until analysis. Heart tissues from the same individual ducks were divided into multiple portions and either snap-frozen in liquid nitrogen or fixed in 4% paraformaldehyde or 2.5% glutaraldehyde. Snap-frozen samples were stored at −80°C for biochemical assays, molecular assays, and proteomic analysis. Samples fixed in 4% paraformaldehyde were kept at room temperature for paraffin embedding, sectioning, and hematoxylin and eosin (H&E) staining, whereas samples fixed in 2.5% glutaraldehyde were stored at 4°C for transmission electron microscopy. Therefore, serum, histological, proteomic, and molecular analyses were performed on aliquots or tissue portions derived from the same set of individual ducks, with pooling used only for proteomic analysis as specified below. All procedures were approved by the Animal Ethics Committee of Hainan Academy of Agricultural Sciences (Approval No. HNXMSY-20250306).

### Culture and heat-treatment of primary duck cardiomyocytes

Primary duck cardiomyocytes were isolated using a protocol adapted, with appropriate modifications, from a previously published method for primary chicken hepatocyte isolation. Briefly, hearts from 18-day-old non–specific-pathogen-free (non-SPF) duck embryos were collected and minced into approximately 1 mm³ fragments. The tissue was digested three times at 37°C with type I collagenase (0.1 mg/mL). Cardiomyocytes were then enriched by differential adhesion. Cells were seeded in Dulbecco’s modified Eagle’s medium (DMEM) supplemented with 20% fetal bovine serum (FBS, v/v) and 1% penicillin–streptomycin (v/v) and maintained at 37°C in a humidified incubator with 5% CO₂. At ∼80% confluence, cells were transferred to a 40°C incubator (5% CO₂) and exposed to heat stress for 0, 1, 2, or 3 h.

### Proteomics analysis and data processing

Fifty milligrams of cardiac tissue was collected from each duck. Equal amounts of tissue from four ducks were pooled to generate one composite biological replicate; thus, five pooled biological replicates were prepared for each group (n = 5 pooled samples per group, 10 pooled samples in total). Proteomic profiling was performed by Guangzhou Gene Denovo Bio-Technology Co., Ltd.

Protein extraction and digestion were carried out using the iST Sample Preparation Kit (PreOmics, Germany) according to the manufacturer’s instructions. Briefly, an appropriate amount of protein was mixed with 50 μL lysis buffer and incubated at 95°C with shaking at 1,000 rpm for 10 min. After cooling to room temperature, trypsin digestion buffer was added and the mixture was incubated at 37°C with shaking at 500 rpm for 2 h. The reaction was stopped by adding stop buffer. Peptides were desalted using an iST cartridge and eluted twice with 100 μL elution buffer. Eluates were vacuum-dried and stored at −80°C until analysis.

Lyophilized peptides were reconstituted in 0.1% formic acid and analyzed by LC–MS/MS on an UltiMate 3000 system (Thermo Fisher Scientific) coupled to a timsTOF Pro2 mass spectrometer (Bruker Daltonics). Peptides were separated on an AUR3-15075 C18 analytical column (15 cm × 75 μm, 1.7 μm, 120 Å) at 50°C and 400 nL/min using a 60-min gradient. Mobile phase B consisted of 80% acetonitrile containing 0.1% formic acid. The gradient was programmed as follows: 4–28% B over 25 min, 28–44% B over 10 min, 44–90% B over 10 min, and a 7-min hold at 90% B, followed by re-equilibration at 4% B for 8 min. Data were acquired in diaPASEF mode (Meier et al., 2020) over an m/z range of 349–1229 with a 40-Da isolation window. For PASEF MS/MS, collision energy was linearly decreased with ion mobility from 59 eV (1/K0 = 1.6 V/cm²) to 20 eV (1/K0 = 0.6 V/cm²).

Raw DIA data were processed in Spectronaut 18 (Biognosys) using the default BGS Factory Settings. Searches were performed against the UniProt Cairina moschata FASTA database (UniProt-Cairina_moschata_8855.fasta). Trypsin was specified as the protease, with cysteine carbamidomethylation as a fixed modification and methionine oxidation as a variable modification. iRT-based automatic retention-time calibration was enabled, and retention-time and mass-extraction windows were applied. A decoy database was generated using a mutation-based strategy. Protein identifications were filtered at ≤1.0% false discovery rate (FDR) at both the precursor and protein levels. Label-free quantification was performed using the MaxLFQ algorithm (Cox et al., 2014) on peptides with FDR < 1.0%, followed by local normalization.

### Bioinformatic analyses

Principal component analysis (PCA) was performed in R (Ihaka and Gentleman, 1996) based on protein abundance profiles. Variance decomposition was used to identify the major sources of variability, thereby evaluating sample similarity and within-group reproducibility. Differentially expressed proteins (DEPs) were identified using Student’s t-test, followed by Benjamini–Hochberg FDR correction for multiple testing (Benjamini and Hochberg, 1995). Significance was defined as an absolute fold change (FC) > 1.5 and adjusted P < 0.05. Volcano plots were generated with the ggplot2 package by plotting log2(FC) versus −log10(Pvalue) to visualize the distribution of DEPs. Heatmaps were produced using the gplots package with hierarchical clustering of Z-score–normalized DEP abundances to reveal expression patterns across conditions. Gene Ontology (GO) enrichment was conducted with the clusterProfiler package across the Biological Process (BP), Cellular Component (CC), and Molecular Function (MF) categories, and significance was assessed using a hypergeometric test followed by Benjamini–Hochberg correction (adjusted P ≤ 0.05). Kyoto Encyclopedia of Genes and Genomes (KEGG) pathway enrichment was performed using KEGG annotations, with significantly enriched pathways identified by a hypergeometric test followed by Benjamini–Hochberg correction (adjusted P ≤ 0.05).

### Hematoxylin and eosin (H&E) staining

Heart tissues fixed in 4% paraformaldehyde were trimmed into blocks (1.5 cm × 1.0 cm × 0.3 cm) and processed using standard paraffin-embedding procedures. Samples were dehydrated through a graded ethanol series (70%, 80%, 90%, 95%, 100%, and 100%; 1 h each), cleared in xylene for 30 min, and infiltrated with molten paraffin for 3 h before embedding. Paraffin blocks were sectioned on a microtome at 4–8 μm. Sections were mounted, flattened, and oven-dried for subsequent staining. H&E staining was performed according to the manufacturer’s instructions (Solarbio, Beijing, China), and myocardial histopathology was examined under a light microscope.

### Transmission electron microscopy (TEM)

Fresh myocardial tissue was immersed in 2.5% glutaraldehyde at 4°C overnight. After fixation, samples were rinsed three times in 0.1 M phosphate buffer (15 min each) and post-fixed in 1% osmium tetroxide at room temperature for 1–2 h, followed by three additional rinses in phosphate buffer. Tissues were dehydrated through a graded ethanol series (30%, 50%, 70%, 80%, 90%, and 95%; 15 min each), then treated twice with absolute ethanol (20 min each) and twice with acetone (20 min each). Samples were infiltrated sequentially with resin/acetone mixtures (1:1, v/v, 1 h; 3:1, v/v, 3 h) and finally with pure resin at room temperature overnight. Specimens were embedded and polymerized at 70°C overnight. Ultrathin sections (70–90 nm) were cut using a Leica EM UC7 ultramicrotome, stained with uranyl acetate and lead citrate (5 min each), air-dried, and examined using a JEOL JEM-1200EX transmission electron microscope.

### Measurement of myocardial injury–associated enzymes

Creatine kinase MB (CK-MB; Catalog No. BP04709, Bpelisa, China) and lactate dehydrogenase (LDH; Catalog No. A020-2, Njjcbio, China) levels were measured in duck serum and cell-culture supernatants using commercially available assay kits. All procedures were performed according to the manufacturers’ instructions.

### Measurement of oxidative stress and ferroptosis-related markers

In myocardial tissues, reactive oxygen species (ROS; Catalog No. G0163W, Geruisi-bio, China), malondialdehyde (MDA; Catalog No. A003-1, Njjcbio, China), glutathione (GSH; Catalog No. A006-2-1, Njjcbio, China), glutathione peroxidase 4 (GPX4; Cat. No. EM1964, FineTest, China), 4-hydroxynonenal (4-HNE; Cat. No. EU0187, FineTest, China), and ferrous iron (Fe²⁺; Cat. No. BC5415, Solarbio, China) were measured using the corresponding commercial kits. In primary cardiomyocytes, GSH, MDA, and GPX4 were measured using the corresponding commercial kits. All procedures were performed according to the manufacturers’ instructions.

### Cell viability assay for primary cardiomyocytes

Cell viability of primary duck cardiomyocytes was assessed using the Cell Counting Kit-8 (CCK-8; Catalog No. PF00004, Proteintech, China) according to the manufacturer’s instructions. Briefly, cells were seeded in 96-well plates at 6 × 10³ cells/well in 100 μL medium. After 30 h of culture to allow stable attachment, plates were transferred to a 40°C incubator (5% CO₂) for heat-stress exposure for 0, 1, 2, or 3 h. Subsequently, 10 μL CCK-8 reagent was added to each well, and plates were incubated in the dark at 37°C with 5% CO₂ for 1.5 h. Absorbance at 450 nm was measured immediately using a microplate reader. Relative cell viability was calculated using the absorbance values of experimental wells after blank subtraction and normalized to the 0-h control.

### Western blotting

Myocardial tissues or primary duck cardiomyocytes were lysed in RIPA buffer, and lysates were centrifuged to collect the supernatant. Total protein concentration was determined using a bicinchoninic acid (BCA) protein assay kit (Catalog No. E112-01, Vazyme, China). Proteins were denatured, separated by sodium dodecyl sulfate–polyacrylamide gel electrophoresis (SDS–PAGE), and transferred onto polyvinylidene difluoride (PVDF) membranes. Membranes were washed with 1 × PBST, blocked with 5% (w/v) nonfat milk, and incubated overnight at 4°C with the appropriate primary antibodies. After washing, membranes were incubated with horseradish peroxidase (HRP)–conjugated secondary antibodies at room temperature for 1 h. Signals were developed using a high-sensitivity enhanced chemiluminescence (ECL) substrate (Catalog No. BL520A, Biosharp, China) and imaged with an automated chemiluminescence imaging system.

Primary antibodies were as follows: β-actin (Catalog No. 66009-1-Ig, Proteintech, China), p-AMPK (Catalog No. 2535, Cell Signaling Technology, USA), AMPK (Catalog No. 10929-2-AP, Proteintech, China), TFR1 (Catalog No. 10084-2-AP, Proteintech, China), ACSL4 (Catalog No. 22401-1-AP, Proteintech, China), FTH1 (Catalog No. 83428-1-RR, Proteintech, China), SLC7A11 (Catalog No. 26864-1-AP, Proteintech, China), GPX4 (Catalog No. 30388-1-AP, Proteintech, China), NCOA4 (Catalog No. 83394-4-RR, Proteintech, China), p-ULK1 (Catalog No. 80218-1-RR, Proteintech, China), and ULK1 (Catalog No. 20986-1-AP, Proteintech, China).

### Statistical analysis

All data were analyzed using SPSS Statistics v25.0 (IBM, USA). Quantitative results are presented as the mean ± standard error of the mean (SEM). Comparisons between two groups were performed using an independent-samples t-test, and differences were considered statistically significant at P < 0.05. For proteomic differential-expression and enrichment analyses, multiple-testing correction was applied as described in the Bioinformatic analyses section.

## Results

### Heat stress induces pathological injury and functional impairment in myocardial tissue

**Fig. 1:**
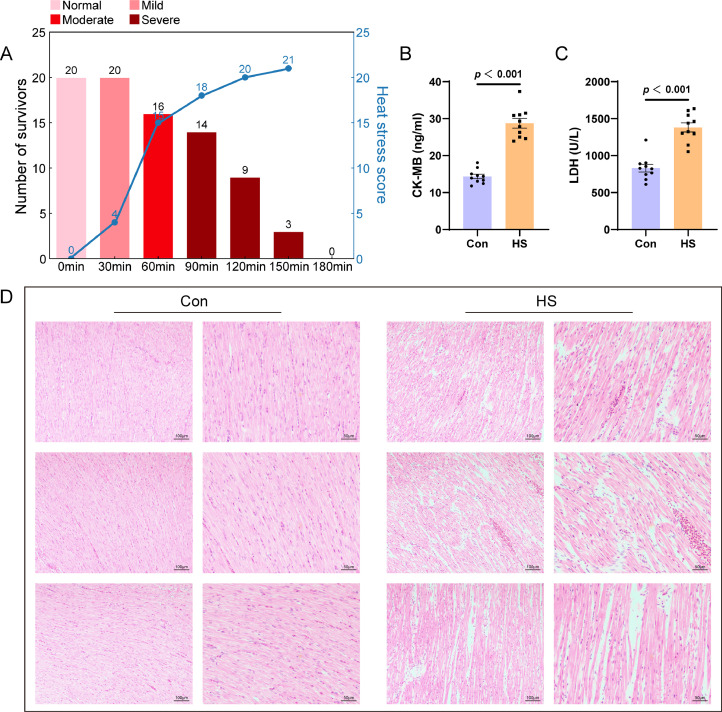
Acute heat stress causes myocardial injury in Jiaji ducks. (A) Survival and heat-stress behavioral scores over time during heat exposure. (B) Serum CK-MB activity in the control (Con) and heat-stress (HS) groups. (C) Serum LDH activity in the Con and HS groups. (D) Representative H&E-stained myocardial sections. Data are presented as mean ± SEM. Groups were compared using an independent-samples *t*-test.

Heat-stress exposure caused rapid physiological deterioration and cardiac dysfunction in ducks. As shown in Fig. 1A, survival declined progressively with increasing duration of heat stress, whereas heat-stress behavioral scores increased over time; by 180 min, all heat-stressed ducks had reached the terminal outcome. Serum collected ante-mortem from the wing vein showed significantly higher CK-MB and LDH activities in the heat-stress group (HS) than in the control group (Con) (both P < 0.001; Fig. 1B, C). Hematoxylin and eosin (H&E) staining showed that myocardial fibers in the Con group were regularly arranged with compact architecture and no obvious pathological lesions. In contrast, HS hearts exhibited pronounced myofiber disorganization, fiber disruption, and enlarged interstitial spaces (Fig. 1D), indicating severe myocardial structural injury.

### Heat stress induces mitochondrial injury and ultrastructural alterations in the myocardium

**Fig. 2:**
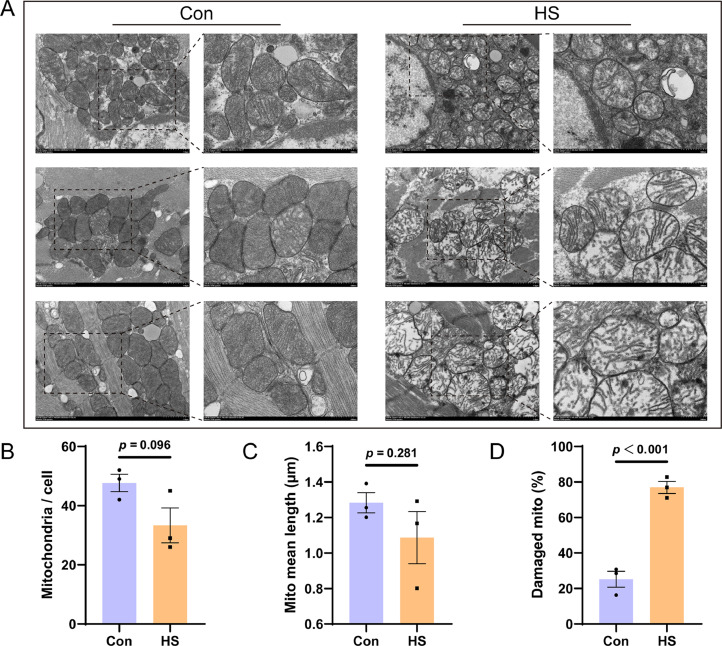
Transmission electron microscopy shows heat stress–induced mitochondrial ultrastructural injury in the myocardium. (A) Representative TEM images of myocardial mitochondria in the Con and HS groups. (B) Mitochondrial number per field. (C) Mean mitochondrial diameter. (D) Proportion of damaged mitochondria. Data are presented as mean ± SEM. Groups were compared using an independent-samples *t*-test.

To characterize subcellular damage caused by heat stress, myocardial ultrastructure was examined by transmission electron microscopy (TEM). As shown in Fig. 2A, mitochondria in cardiomyocytes from the control group (Con) displayed normal morphology with intact cristae, whereas those in the heat-stress group (HS) were markedly shrunken and exhibited disrupted, reduced, or absent cristae. These alterations suggest impairment of mitochondrial inner-membrane architecture, which is closely related to respiratory-chain function and intracellular redox homeostasis.

Quantitative analysis showed a modest reduction in mitochondrial number per field in the HS group, but this difference did not reach statistical significance (*P* = 0.096; Fig. 2B). Mean mitochondrial diameter also trended lower without a significant difference (*P* = 0.281; Fig. 2C). In contrast, the proportion of damaged mitochondria was significantly higher in the HS group than in the Con group (*P* !< 0.001; Fig. 2D).

### Heat stress remodels the myocardial proteome and alters protein expression

**Fig. 3:**
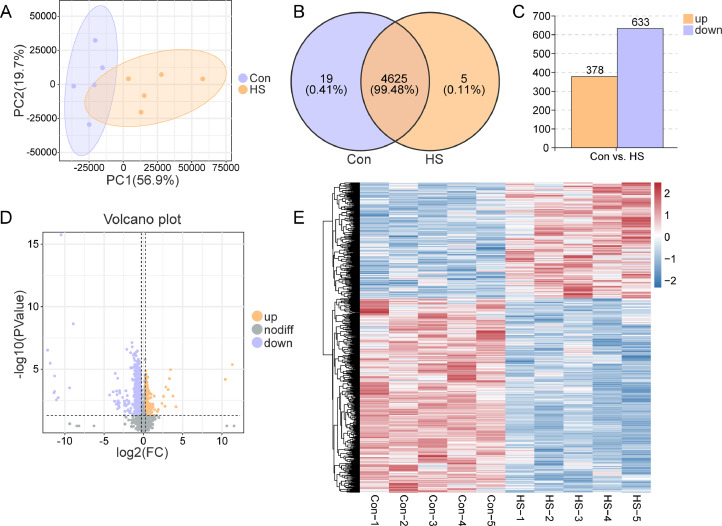
Heat stress remodels the myocardial proteome in Jiaji ducks (proteomics). (A) Principal component analysis (PCA) showing separation between the Con and HS groups. (B) Overlap of proteins detected in the two groups. (C) Numbers of upregulated and downregulated proteins. (D) Volcano plot of differentially expressed proteins (|FC| > 1.5, P < 0.05). (E) Hierarchical clustering heatmap of differentially expressed proteins.

To assess the impact of heat stress on myocardial protein expression, we performed quantitative proteomic profiling. Principal component analysis (PCA) showed clear separation between the control (Con) and heat-stress (HS) groups, with PC1 and PC2 explaining 56.9% and 19.7% of the total variance, respectively (Fig. 3A). This pattern indicates that heat stress markedly remodeled the global myocardial proteome and that within-group reproducibility was sufficient to distinguish the two physiological states. In total, 4,625 proteins were detected in both groups (99.48% of all identified proteins), whereas 19 and 5 proteins were uniquely detected in the Con and HS groups, respectively (Fig. 3B). Differential expression analysis identified 1,011 significantly altered proteins, including 378 upregulated and 633 downregulated proteins (Fig. 3C), showing that the heat-stress response involved both activation and suppression of broad protein networks. The distribution of differentially expressed proteins is shown in the volcano plot (Fig. 3D). Consistently, hierarchical clustering of differentially expressed proteins revealed distinct expression patterns between groups (Fig. 3E), further supporting extensive heat stress–induced myocardial proteome reprogramming.

### GO functional annotation and KEGG pathway enrichment of differentially expressed proteins

**Fig. 4:**
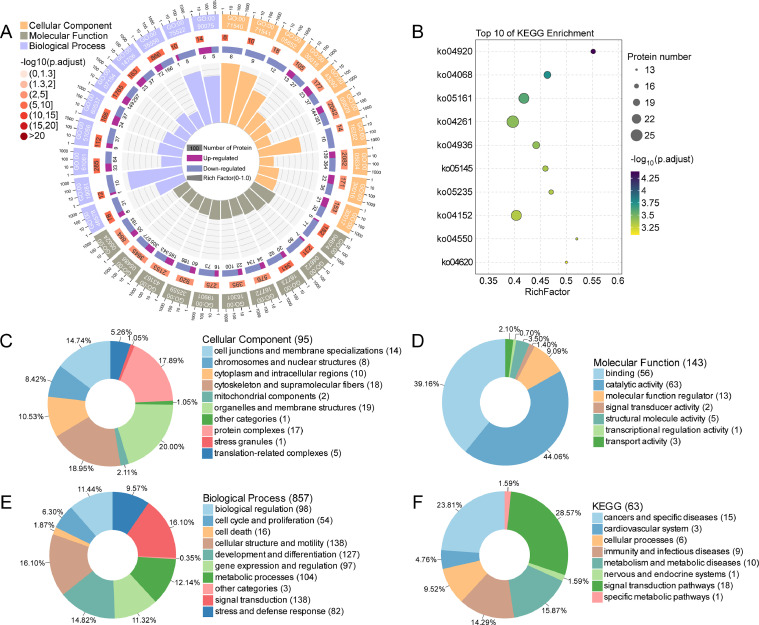
GO and KEGG enrichment analyses of differentially expressed proteins in heat-stressed Jiaji duck myocardium. (A) Gene Ontology (GO) enrichment across Biological Process, Molecular Function, and Cellular Component categories. (B) Kyoto Encyclopedia of Genes and Genomes (KEGG) pathway enrichment. (C–F) Functional classification of enriched terms.

To investigate the biological functions and putative regulatory networks associated with heat stress, we performed Gene Ontology (GO) annotation and Kyoto Encyclopedia of Genes and Genomes (KEGG) pathway enrichment analyses of differentially expressed proteins in myocardial tissue. GO enrichment indicated that these proteins were mainly distributed across the Biological Process (BP), Cellular Component (CC), and Molecular Function (MF) categories. The most enriched BP terms included regulation of Ras protein signal transduction, viral translation, and muscle structure development; enriched MF terms included serine/threonine protein kinase activity and protein kinase activity; and enriched CC terms were dominated by components of the eukaryotic translation initiation factor 3 (eIF3) complex (Fig. 4A). These terms suggest that heat stress affected signal transduction, protein synthesis/translation-related processes, and muscle structural remodeling, all of which are relevant to myocardial adaptation and injury. KEGG analysis further showed enrichment of the adipokine signaling pathway, FoxO signaling pathway, and hepatitis B pathway (Fig. 4B). Notably, the AMPK signaling pathway was also significantly enriched, suggesting activation of energy-stress–related signaling. Functional classification of the enriched terms indicated that the largest functional groups were cell death, stress and defense responses, mitochondrial components, signal transduction pathways, and cancer- and disease-related categories (Fig. 4C–F). These enriched categories are consistent with the histological and ultrastructural evidence of myocardial injury and suggest that mitochondrial stress, cell-death regulation, and compensatory defense responses are major components of the heat-stress proteomic signature.

### Ferroptosis-related enrichment and GPX4-associated network analysis

**Fig. 5:**
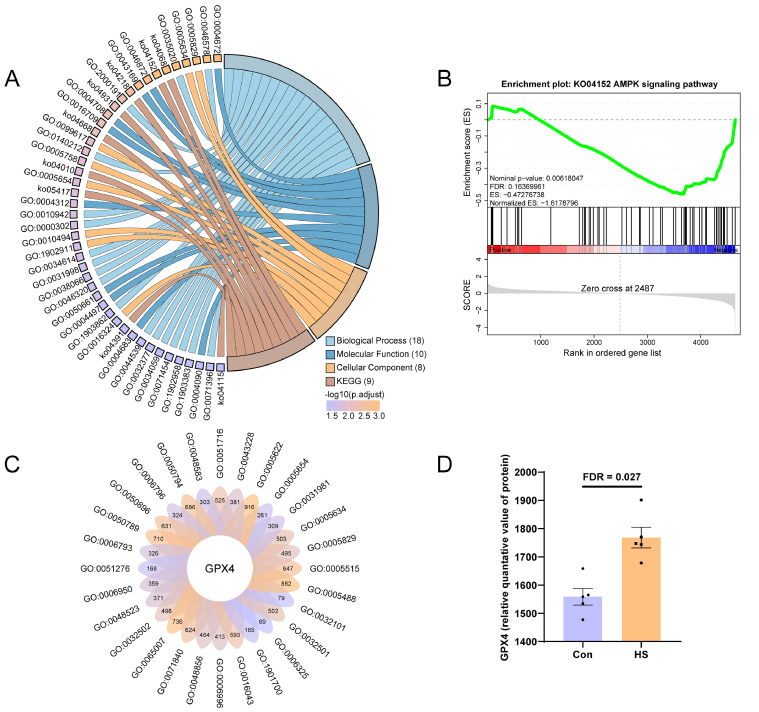
Ferroptosis-related enrichment in heat-stressed Jiaji duck myocardium and GPX4-associated network analysis. (A) Summary of ferroptosis-related GO/KEGG terms enriched among differentially expressed proteins. (B) KEGG enrichment highlighting the AMPK signaling pathway. (C) GPX4-associated functional interaction network. (D) Proteomics-derived GPX4 protein abundance in myocardium from the Con and HS groups. Data are presented as mean ± SEM. Groups were compared using an independent-samples t-test; GPX4 direction was further evaluated by targeted biochemical and western blot assays.

To explore the molecular basis of heat stress–induced myocardial injury, we performed functional enrichment analyses of differentially expressed proteins. As shown in Fig. 5A, these proteins were significantly enriched in ferroptosis-related Gene Ontology (GO) terms, including 18 Biological Process, 8 Cellular Component, and 10 Molecular Function terms, as well as 9 KEGG pathways. This enrichment indicates that ferroptosis-associated processes were represented within the myocardial proteomic response to heat stress. KEGG enrichment also highlighted the AMPK signaling pathway (Fig. 5B), which is closely linked to energy-stress signaling and autophagy regulation, suggesting a potential connection between energy imbalance, autophagy-related processes, and ferroptosis-associated injury. In network analyses, glutathione peroxidase 4 (GPX4), a core anti-ferroptotic enzyme, was broadly connected to glutathione metabolism, oxidative-stress responses, and cell-death processes (Fig. 5C), supporting its role as a regulatory hub in this network. Proteomic quantification showed a significant increase in GPX4 abundance in myocardial tissue from the HS group compared with the Con group (FDR = 0.027; Fig. 5D). Given the central role of GPX4 in antioxidant defense and ferroptosis regulation, this finding suggested a disturbance of the GPX4-related antioxidant network and required further targeted validation.

### Biochemical validation of heat stress–induced cardiac ferroptosis

**Fig. 6:**
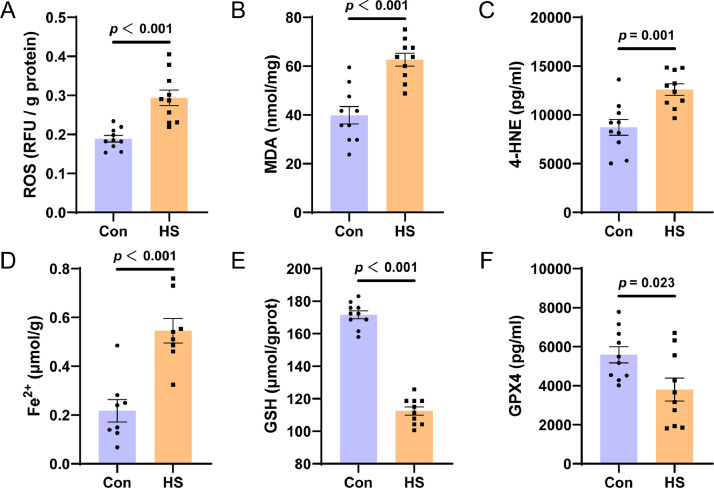
Heat stress alters ferroptosis-associated biochemical markers in Jiaji duck hearts. (A) ROS levels. (B) MDA content. (C) 4-HNE content. (D) Fe²⁺ content. (E) GSH content. (F) GPX4 protein abundance in myocardial tissue. Data are presented as mean ± SEM. Groups were compared using an independent-samples *t*-test.

To validate the ferroptosis-associated signature suggested by the proteomic analysis, we quantified key biochemical markers of ferroptosis in myocardial tissue. Compared with the control group (Con), myocardial ROS levels were significantly higher in the heat-stress group (HS) (P < 0.001; Fig. 6A), indicating pronounced oxidative stress. Consistently, the lipid peroxidation markers malondialdehyde (MDA) and 4-hydroxynonenal (4-HNE) were increased (P < 0.001 and P = 0.001, respectively; Fig. 6B, C), supporting enhanced lipid peroxidation in the myocardium. Indicators of iron metabolism further showed a marked elevation of ferrous iron (Fe²⁺) in the HS group (P < 0.001; Fig. 6D), suggesting disruption of intracellular iron homeostasis. In parallel, glutathione (GSH) levels were significantly reduced after heat stress (P < 0.001; Fig. 6E), and GPX4 protein abundance was decreased (P = 0.023; Fig. 6F). These targeted biochemical results were considered more directly informative for the direction of GPX4 change and the functional status of the GSH–GPX4 antioxidant axis than the discovery-level proteomic signal. Together, iron accumulation, impairment of the GSH–GPX4 antioxidant axis, and increased ROS-driven lipid peroxidation are consistent with a ferroptosis-like phenotype.

### Heat stress modulates key ferroptosis-related proteins in myocardial tissue

**Fig. 7:**
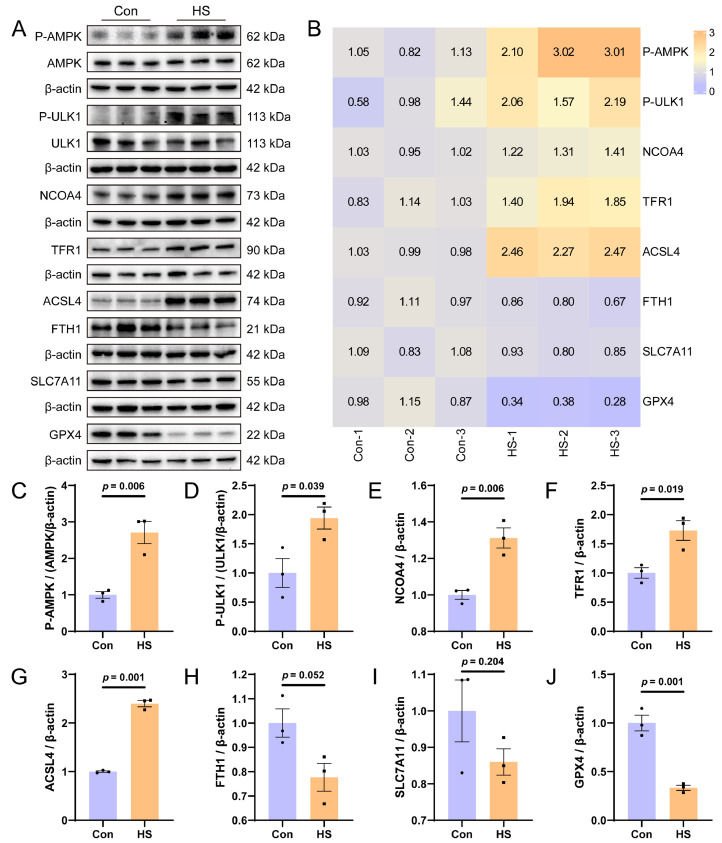
Heat stress modulates AMPK/ULK1 signaling and ferroptosis-related proteins in Jiaji duck myocardium. Representative western blots and densitometric quantification of p-AMPK, AMPK, p-ULK1, ULK1, TFR1, NCOA4, FTH1, ACSL4, SLC7A11, and GPX4 in the Con and HS groups; β-actin served as the loading control. Heat stress increased p-AMPK, p-ULK1, TFR1, NCOA4, and ACSL4, whereas FTH1 showed a decreasing trend and SLC7A11 and GPX4 were reduced. Uncropped membrane scans are provided in Additional file 2. Densitometry data are presented as mean ± SEM. Groups were compared using an independent-samples *t*-test.

To further examine whether heat stress alters ferroptosis-related signaling at the protein level, we used western blotting to quantify changes in key regulators in myocardial tissue (Fig. 7). Compared with the control group (Con), the AMPK/ULK1 pathway showed increased activation in the heat-stress group (HS), as indicated by higher phosphorylation levels of AMPK and ULK1 (p-AMPK and p-ULK1). Proteins involved in iron handling also shifted toward an iron-loading profile: transferrin receptor 1 (TFR1) and nuclear receptor coactivator 4 (NCOA4) were upregulated, whereas ferritin heavy chain 1 (FTH1) showed a decreasing trend. This pattern suggests increased iron uptake together with reduced iron-buffering capacity and possible ferritinophagy-mediated iron release. In addition, acyl-CoA synthetase long-chain family member 4 (ACSL4), a lipid peroxidation–promoting enzyme, was increased after heat stress, indicating higher susceptibility of membrane phospholipids to peroxidation. Conversely, components of the anti-ferroptotic defense system were reduced, with a slight decrease in SLC7A11 and a significant reduction in GPX4. These western blot results therefore support the biochemical findings and indicate that heat stress modulates a coordinated ferroptosis-related regulatory network in myocardial tissue.

### In vitro evidence indicates that heat stress directly promotes ferroptosis-like injury in primary cardiomyocytes

**Fig. 8:**
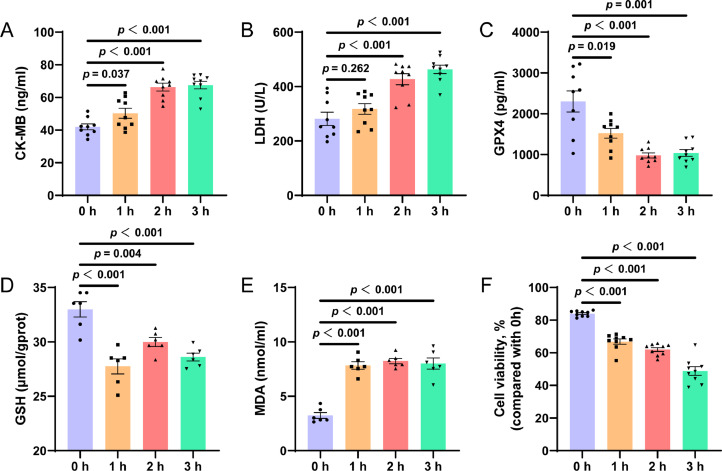
In vitro heat stress induces cardiomyocyte injury and ferroptosis-associated biochemical changes. (A) CK-MB release into culture supernatants after heat stress for 0, 1, 2, or 3 h. (B) LDH release into culture supernatants after heat stress for 0, 1, 2, or 3 h. (C) GPX4 protein abundance. (D) Intracellular GSH content. (E) Intracellular MDA content. (F) Cell viability measured by CCK-8. Data are presented as mean ± SEM. Each time point was compared with the 0-h control using independent-samples *t*-tests.

To determine whether heat stress directly injures cardiomyocytes and induces ferroptosis-associated changes, primary duck cardiomyocytes were exposed to heat stress for 0, 1, 2, or 3 h, followed by multiparametric assessment. The myocardial injury markers CK-MB and LDH increased in the culture supernatant in a time-dependent manner (Fig. 8A, B), indicating compromised membrane integrity. With increasing exposure duration, GPX4 protein abundance (Fig. 8C) and intracellular glutathione (GSH) levels (Fig. 8D) decreased, whereas malondialdehyde (MDA) levels increased (Fig. 8E), consistent with enhanced lipid peroxidation and impaired antioxidant defense. Cell viability also declined significantly with prolonged heat stress (Fig. 8F). Overall, these in vitro results mirror the in vivo findings and support heat stress as a direct driver of ferroptosis-like changes in cardiomyocytes.

### In vitro western blotting reveals heat stress–dependent modulation of the ferroptosis regulatory network

**Fig. 9:**
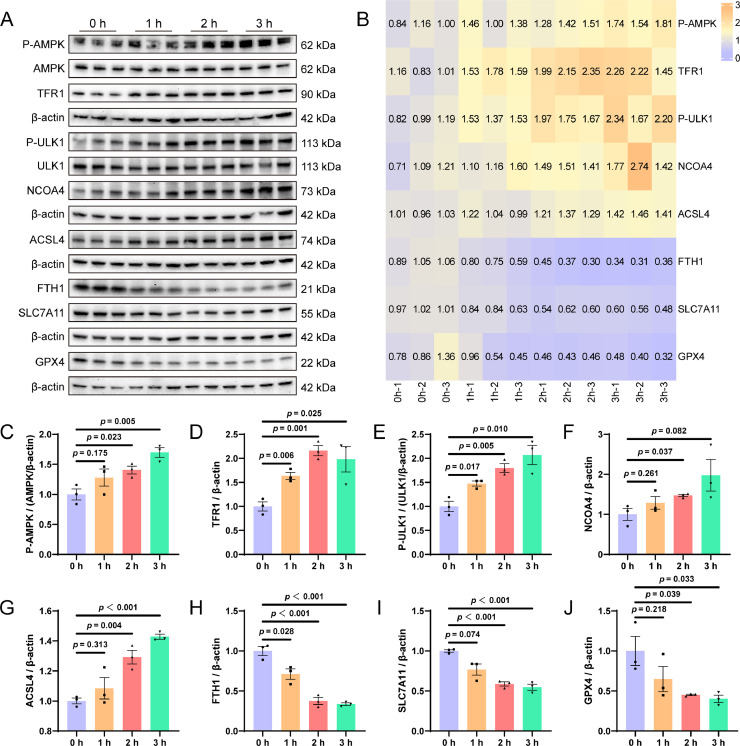
Time-dependent effects of in vitro heat stress on AMPK/ULK1 signaling and ferroptosis-related proteins in primary cardiomyocytes. Primary cardiomyocytes were exposed to 40°C for 0, 1, 2, or 3 h. Protein levels of p-AMPK, AMPK, p-ULK1, ULK1, TFR1, NCOA4, FTH1, ACSL4, SLC7A11, and GPX4 were assessed by western blotting, with β-actin as the loading control. Heat stress increased p-AMPK and p-ULK1 and upregulated TFR1, NCOA4, and ACSL4, whereas FTH1 trended downward and SLC7A11 and GPX4 decreased over time. Uncropped membrane scans are provided in Additional file 2. Densitometry data are presented as mean ± SEM. Each time point was compared with the 0-h control using independent-samples *t*-tests.

To evaluate whether heat stress directly modulates ferroptosis-related pathways in vitro, we performed western blotting to quantify key regulatory proteins in primary cardiomyocytes after 0, 1, 2, or 3 h of heat exposure (Fig. 9). Heat stress increased activation of the AMPK/ULK1 axis, as reflected by time-dependent increases in AMPK and ULK1 phosphorylation (p-AMPK and p-ULK1). Proteins involved in iron handling were also altered: TFR1 progressively increased with exposure duration, NCOA4 was upregulated after 2 h, and FTH1 showed a decreasing trend. In addition, ACSL4 levels were elevated at 2 and 3 h, consistent with enhanced susceptibility to lipid peroxidation. Conversely, anti-ferroptotic defenses were reduced, with SLC7A11 downregulated at 2 and 3 h and GPX4 decreasing in a time-dependent manner. Overall, these protein-level changes in primary cardiomyocytes closely mirrored those observed in myocardial tissue in vivo, supporting a direct effect of heat stress that is less confounded by systemic influences (e.g., neurohumoral regulation).

## Discussion

The heart is essential for maintaining systemic perfusion and metabolic homeostasis and therefore depends on sustained, high-intensity contraction coupled with stable electrophysiological and metabolic regulation. When heat stress disrupts redox balance and precipitates energetic stress, cardiac function is particularly prone to decompensation (Li et al., 2023b; Roths et al., 2024; Yao et al., 2025). Accordingly, the heart is among the organs most vulnerable to heat stress–induced injury in poultry. Consistent with previous poultry studies showing that heat exposure increases cardiac injury markers and induces myocardial oxidative injury, apoptosis, or autophagy-related responses (Xu et al., 2019; Yao et al., 2025), acute heat stress in the present study caused severe myocardial injury in Jiaji ducks within a short period. Survival declined rapidly as exposure time increased, behavioral heat-stress scores rose progressively, and all heat-stressed ducks reached the terminal outcome by 180 min. In parallel, serum CK-MB and LDH levels increased significantly, and H&E staining revealed myofiber disorganization, fragmentation, and enlarged interstitial spaces. Collectively, these findings indicate that acute heat stress compromises cardiomyocyte membrane integrity and promotes structural injury of the myocardium.

A defining feature of heat stress–induced organ injury is a rapid disruption of intracellular homeostasis, characterized by increased energy demand and redox imbalance. Mitochondria are among the earliest and most vulnerable targets of heat stress (Huang et al., 2015; Slimen et al., 2014). In broiler chickens, heat stress has been reported to impair mitochondrial function and promote oxidative injury, highlighting the central role of mitochondria in avian thermal stress responses (Huang et al., 2015). Similar mitochondrial vulnerability has also been observed in mammalian myocardium after acute environmental heat exposure (Roths et al., 2024). Cardiomyocytes are highly dependent on mitochondrial function because ATP is generated primarily through oxidative phosphorylation and the myocardium operates under sustained metabolic demand. When mitochondria are damaged, reduced ATP production and increased reactive oxygen species (ROS) generation can reinforce each other, creating a self-amplifying cycle of injury (Suleiman et al., 2001; Tsutsui et al., 2011). In this study, TEM showed that myocardial mitochondria in the heat-stressed group were shrunken and exhibited reduced or absent cristae. Although mitochondrial number and mean diameter did not differ significantly between groups, the proportion of damaged mitochondria increased markedly, suggesting that heat stress primarily compromises mitochondrial quality rather than quantity. Cristae disruption reflects damage to the inner membrane architecture that supports respiratory-chain complexes, which can decrease electron transport efficiency and increase electron leakage, thereby promoting ROS production. Excess ROS, in turn, accelerates lipid peroxidation and compromises membrane integrity (Zorov et al., 2014). Together with the increased serum CK-MB and LDH levels observed here, these findings support a mechanistic link between mitochondrial ultrastructural injury, heightened oxidative stress, and myocardial membrane damage.

Previous proteomic studies have shown that heat stress can remodel myocardial protein expression profiles and alter pathways related to nucleic acid metabolism, mitochondrial function, and stress responses in chicken myocardial cells or cardiac tissues (Qin et al., 2024; Wang et al., 2023; Zhu et al., 2025). Consistent with these reports, our proteomic data indicate that acute heat stress markedly altered the myocardial proteome of Jiaji ducks. PCA demonstrated clear separation between control and heat-stressed samples, and more than 1,000 proteins were significantly differentially expressed, indicating a broad molecular response to thermal challenge. GO and KEGG enrichment analyses further suggested that these proteins were overrepresented in mitochondria-related terms, stress/defense responses, signal-transduction pathways, and cell-death categories. Thus, the proteomic changes observed here are not isolated molecular fluctuations but align closely with the histological and ultrastructural evidence of injury. Notably, the AMPK signaling pathway was significantly enriched. Consistently, immunoblotting showed increased phosphorylation of AMPK and ULK1 (p-AMPK and p-ULK1) in heat-stressed myocardium. AMPK is a central cellular energy sensor that is activated during energetic stress (e.g., ATP depletion with increased AMP/ATP ratio). Once activated, AMPK can promote autophagy through ULK1, facilitating adaptation to energy limitation and restoration of metabolic homeostasis (Herzig and Shaw, 2018; Kim et al., 2011). In a high-energy–demand tissue such as the myocardium, activation of the AMPK–ULK1 axis therefore suggests substantial metabolic stress and a heightened requirement for turnover and repair (Feng et al., 2018; Jiang et al., 2021). Coupled with the mitochondrial ultrastructural injury observed here, these data support the interpretation that AMPK–ULK1 activation represents a compensatory response to mitochondrial dysfunction and impaired energy supply.

Ferroptosis is an iron-dependent form of regulated cell death driven by lipid peroxidation. It is characterized by persistent peroxidation of membrane phospholipids, culminating in loss of membrane integrity (Dixon and Olzmann, 2024). In the canonical regulatory axis, cells import cystine via SLC7A11 (system Xc⁻) to support synthesis of reduced glutathione (GSH). As the essential reducing cofactor for GPX4, GSH enables GPX4 to detoxify phospholipid hydroperoxides and thereby suppress lipid peroxidation chain reactions (Dixon and Olzmann, 2024; Zhou et al., 2024). When iron accumulates and GSH is depleted and/or GPX4 activity is impaired, lipid peroxidation accelerates, leading to accumulation of end products such as MDA and 4-HNE and ultimately causing irreversible membrane damage (Yang et al., 2019; Dixon and Olzmann, 2024; Li et al., 2024). In the present study, heat stress significantly increased ROS levels in Jiaji duck myocardium, accompanied by elevations in MDA and 4-HNE and an increase in Fe²⁺. In parallel, GSH levels declined markedly, and targeted biochemical and western blot assays showed reduced GPX4 abundance, together forming a pattern consistent with ferroptosis-like injury. This finding extends previous evidence that ferroptosis contributes to mammalian myocardial injury and cardiovascular disease (Fang et al., 2020; Wu et al., 2021; Zhao et al., 2025) to an avian heat-stress context. Notably, GPX4 appeared upregulated in the proteomic dataset but downregulated by western blotting and biochemical assays. We consider the targeted biochemical and western blot results more reliable for determining the direction of GPX4 change in this study because they directly assayed GPX4 abundance in non-pooled validation samples and were consistent across both in vivo and in vitro experiments. The discrepancy may reflect differences in platform principles: proteomics quantified peptide-level abundance in pooled samples, whereas antibody-based assays detected the intact or epitope-accessible protein; moreover, post-translational modifications, oxidation, proteolytic processing, or altered antibody epitope accessibility may influence cross-platform consistency. Although the precise cause of this discrepancy was not further dissected, the pronounced GSH depletion, increased lipid peroxidation products, Fe²⁺ accumulation, and GPX4 decrease in targeted assays collectively support impairment of the GPX4-mediated anti-ferroptotic defense system after heat stress.

Disrupted iron homeostasis is a key prerequisite for ferroptosis. In this study, protein-expression analyses provide a mechanistic explanation for the heat stress–associated accumulation of ferrous iron in cardiomyocytes. After heat stress, TFR1 was upregulated, consistent with enhanced iron uptake (Park and Chung, 2019). In contrast, FTH1 showed a decreasing trend, suggesting reduced iron storage and buffering capacity (Cui et al., 2024). In parallel, NCOA4, a central mediator of ferritinophagy, increased, implying enhanced ferritin degradation and release of stored iron into the labile iron pool, thereby facilitating iron-dependent oxidative reactions (Ohshima et al., 2022). Moreover, ACSL4 was upregulated, suggesting increased incorporation of polyunsaturated fatty acids into membrane phospholipids and heightened susceptibility to lipid peroxidation (Pei et al., 2021; Zhang et al., 2022). Meanwhile, downregulation of the cystine transporter SLC7A11 likely limited cystine uptake, reducing GSH synthesis and further weakening the GSH–GPX4 anti-ferroptotic defense axis (Fang et al., 2020; Li et al., 2023c). Collectively, these changes indicate that heat stress promotes iron loading, diminishes iron buffering, and increases lipid peroxidation substrate availability; together with impaired antioxidant capacity, these conditions favor phospholipid peroxidation and a ferroptosis-like phenotype in cardiomyocytes. Notably, increased AMPK/ULK1 phosphorylation coincided with NCOA4 upregulation, suggesting that activation of energy-stress signaling is accompanied by enhanced autophagy-related processes. Because ferritinophagy is a key route by which stored iron is mobilized into the labile iron pool, this pathway may represent an important coupling node between energy stress and ferroptosis during heat stress (Qin et al., 2021; Wang et al., 2025).

In vitro experiments using primary cardiomyocytes from Jiaji ducks further supported the mechanistic relationships proposed above. With increasing heat-stress duration, CK-MB and LDH release into the culture supernatant increased in a time-dependent manner, accompanied by a significant decline in cell viability. In parallel, intracellular GSH levels and GPX4 protein abundance decreased, whereas MDA levels increased, indicating impaired antioxidant capacity and enhanced lipid peroxidation. Heat stress also increased activation of the AMPK/ULK1 axis, reflected by elevated p-AMPK and p-ULK1. Consistently, TFR1, NCOA4, and ACSL4 were upregulated, whereas FTH1, SLC7A11, and GPX4 were downregulated, aligning with iron loading and heightened susceptibility to lipid peroxidation. These in vitro trends mirrored those observed in myocardial tissue in vivo. Because the cell-culture model is less influenced by systemic confounders (e.g., neurohumoral regulation and blood-flow redistribution), the findings support heat stress as a direct driver of energetic stress, iron dyshomeostasis, and lipid peroxidation in cardiomyocytes. Under conditions of a weakened GSH–GPX4 defense axis, these changes are consistent with a ferroptosis-like injury phenotype. Together with TEM evidence of mitochondrial ultrastructural disruption and the observed increase in ROS, the data support a model in which mitochondrial injury–associated oxidative stress synergizes with iron accumulation and increased peroxidation-prone membrane substrates to overwhelm antioxidant defenses, culminating in irreversible cardiomyocyte damage and the severe myocardial structural and functional impairment reported here.

Several limitations should be acknowledged. First, the in vivo experiment used an acute lethal heat-stress challenge designed to model severe hot–humid field episodes and acute death risk rather than chronic sublethal heat exposure. Therefore, systemic responses such as circulatory failure and multi-organ injury may have contributed to the myocardial phenotype, and ferroptosis should be interpreted as an important associated mechanism rather than the only pathway involved. Second, although all blood samples were collected ante-mortem and tissues were sampled immediately after death to minimize post-mortem artifacts, this model cannot completely exclude terminal systemic effects. Third, in the in vitro validation experiment, ROS, 4-HNE, and Fe²⁺ were not measured because only GSH, MDA, and GPX4 were included in the cell-compatible validation assays available for this study. Finally, intervention experiments using ferroptosis inhibitors or genetic manipulation of GPX4/SLC7A11/NCOA4 will be needed to establish causality more directly.

## Conclusion

This study demonstrates that acute heat stress rapidly induces severe myocardial structural and functional injury in Jiaji ducks, manifested by compromised cardiomyocyte membrane integrity, myofiber disruption and disorganization, and pronounced mitochondrial ultrastructural damage. Mechanistically, heat stress was associated with activation of the AMPK/ULK1 axis, accompanied by perturbations in iron homeostasis and lipid metabolism. These changes favored Fe²⁺ accumulation and lipid peroxidation while depleting intracellular GSH and suppressing the SLC7A11–GPX4 antioxidant defense system in targeted validation assays. Collectively, the in vivo and in vitro findings are consistent with ferroptosis-like cardiomyocyte injury characterized by iron-dependent lipid peroxidation. In primary Jiaji duck cardiomyocytes, heat stress alone elicited comparable ferroptosis-associated molecular changes, supporting a direct cellular effect. Overall, ferroptosis-related regulatory nodes may represent important contributors to the initiation and progression of heat stress–induced myocardial injury in Jiaji ducks, although additional intervention studies are needed to confirm direct causality.

## Ethics approval

All procedures were approved by the Animal Ethics Committee of Hainan Academy of Agricultural Sciences (Approval No. HNXMSY-20250306).

## Data and model availability statement

All data necessary to support the conclusions of this study is either included in the paper. The datasets generated and/or analyzed during the current study are available upon reasonable request from the corresponding author.

## Declaration of generative AI and AI-assisted technologies in the writing process

During the preparation of this manuscript the authors used ChatGPT 5.5 for word editing only to eliminate typos and grammatical errors. The authors then reviewed and edited the content as needed. The authors take full responsibility for the content of the manuscript.

## Financial support statement

This work was supported by the Hainan Provincial Key R&D Program (ZDYF2024XDNY158), the National Key Research and Development Program of China (2023YFD1300303), the China Agriculture Research System (CARS-42-50), and the Hainan Academy of Agricultural Sciences Program (HAAS2025MLXXQ01).

## CRediT authorship contribution statement

**Qijun Liang:** Formal analysis, Data curation, Writing – original draft, Writing – review & editing, Visualization. **Xinli Zheng:** Methodology, Validation, Formal analysis, Writing – review & editing. **Tieshan Xu:** Conceptualization, Resources, Writing – review & editing. **Piaoran Liu:** Validation, Methodology, Investigation. **Yiyong Chen:** Conceptualization, Investigation, Resources. **Zhemin Lin:** Validation, Resources, Writing – review & editing. **Lihong Gu:** Writing – review & editing, Supervision, Project administration, Funding acquisition.

## Disclosures

The authors declare no competing interests.

## Appendix Supplementary materials

Image, application 1

Image, application 2

**Supplementary material:** Supplementary Material for this article can be found at the foot of the online page, in the Appendix section. Image, application 3
