# Supplementary material for: Heat stress promotes ferroptosis in Jiaji duck myocardium by disrupting iron homeostasis and inducing lipid peroxidation
Source: Poult Sci. 2026 Jun 9;105(9):107241. doi: 10.1016/j.psj.2026.107241 (PMC13292588; doi:10.1016/j.psj.2026.107241)
Supplement: Supplementary file 1 [file mmc1.docx]

**Supplementary Tables**

*Heat stress clinical scoring system in Jiaji ducks*

**Table S1.** Clinical scoring criteria for heat stress severity in Jiaji ducks.

| **Clinical indicator** | **0 points** | **1 point** | **2 points** | **3 points** |
| --- | --- | --- | --- | --- |
| **Posture and locomotion** | Normal standing and free movement | Occasionally lies down but can stand up independently | Lies down for extended periods; barely able to walk when driven | Continuously recumbent; unable to stand |
| **Open-mouth breathing** | Breathing stable; mouth closed | Open mouth only after heat exposure or activity | Continuous open-mouth breathing with slightly increased frequency | Severe open-mouth breathing with neck extension |
| **Respiratory rate** | Stable and within the normal range | Slightly increased | Markedly increased with obvious abdominal movement | Extremely rapid with pronounced panting |
| **Wing posture** | Wings held close to the body | Slightly drooping or occasionally spread | Obviously spread and drooping | Fully spread and drooping; unable to retract |
| **Mental status** | Alert and responsive | Slightly depressed but still responsive | Depressed with slow responses | Severely depressed with weak responses to external stimuli |
| **Sleep state** | No abnormal sleepiness | Mild drowsiness but easily aroused | Persistent lethargy; requires strong stimulation to arouse | Comatose state; difficult to arouse |
| **Fecal consistency** | Normally formed feces | Slightly soft; partially formed | Watery diarrhea with minimal formation | Persistent watery diarrhea |

Note. Each clinical indicator was scored from 0 to 3, and the total score was used to classify heat stress severity as follows: 0 ≤ score < 3, normal; 3 ≤ score < 10, mild heat stress; 10 ≤ score < 18, moderate heat stress; and score ≥ 18, severe heat stress.

Source note. The criteria were adapted from chicken heat stress experiments and published literature (doi:10.1016/j.psj.2023.103340; doi:10.1016/j.ijbiomac.2024.135979).

**Table S2.** Summary of clinical scoring results for Jiaji ducks at different heat stress exposure durations.

| **Time point (min)** | **Mild (n/N)** | **Moderate (n/N)** | **Severe (n/N)** | **Mean total score** | **Survivors (n)** | **Cumulative deaths (n)** | **Mortality (%)** |
| --- | --- | --- | --- | --- | --- | --- | --- |
| **0** | 0/20 | 0/20 | 0/20 | 0 | 20 | 0 | 0 |
| **30** | 20/20 | 0/20 | 0/20 | 4 | 20 | 0 | 0 |
| **60** | 0/16 | 16/16 | 0/16 | 15 | 16 | 4 | 20 |
| **90** | 0/14 | 4/14 | 10/14 | 18 | 14 | 6 | 30 |
| **120** | 0/9 | 0/9 | 9/9 | 20 | 9 | 11 | 55 |
| **150** | 0/3 | 0/3 | 3/3 | 21 | 3 | 17 | 85 |
| **180** | — | — | — | — | 0 | 20 | 100 |

Note. Values for heat stress categories are presented as n/N. An em dash indicates that no ducks survived to the corresponding time point and, therefore, no clinical category or mean score could be assigned.
