# Supplementary material for: Heat stress promotes ferroptosis in Jiaji duck myocardium by disrupting iron homeostasis and inducing lipid peroxidation
Source: Poult Sci. 2026 Jun 9;105(9):107241. doi: 10.1016/j.psj.2026.107241 (PMC13292588; doi:10.1016/j.psj.2026.107241)

Figure 7 (A: Con; B: HS)

Phospho-AMPK alpha (Thr172) (40H9) Rabbit mAb (Cell Signaling, #2535)

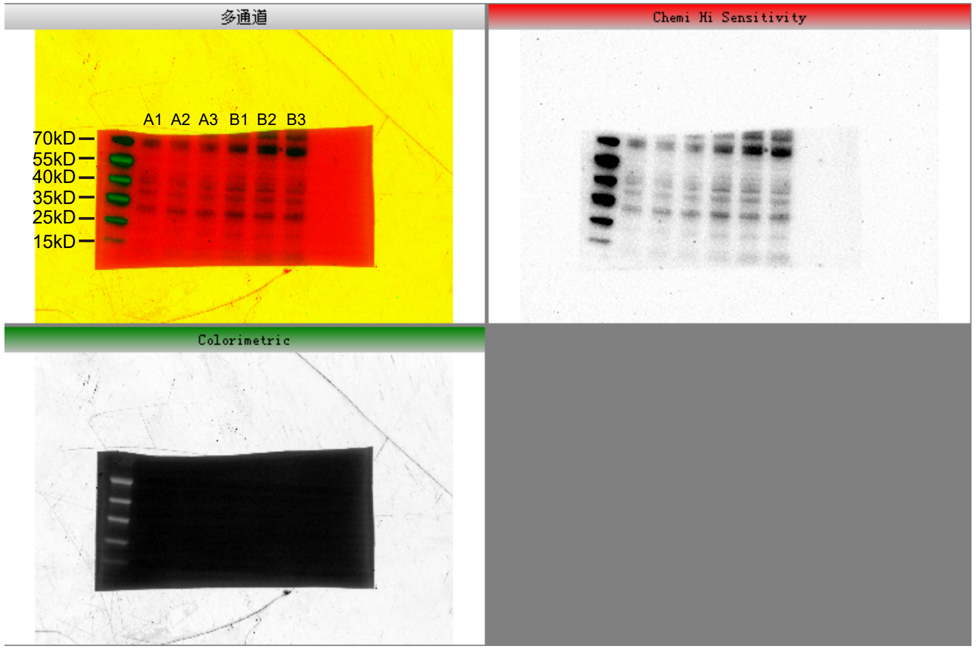

AMPK Alpha Polyclonal antibody (Proteintech, #10929-2-AP)

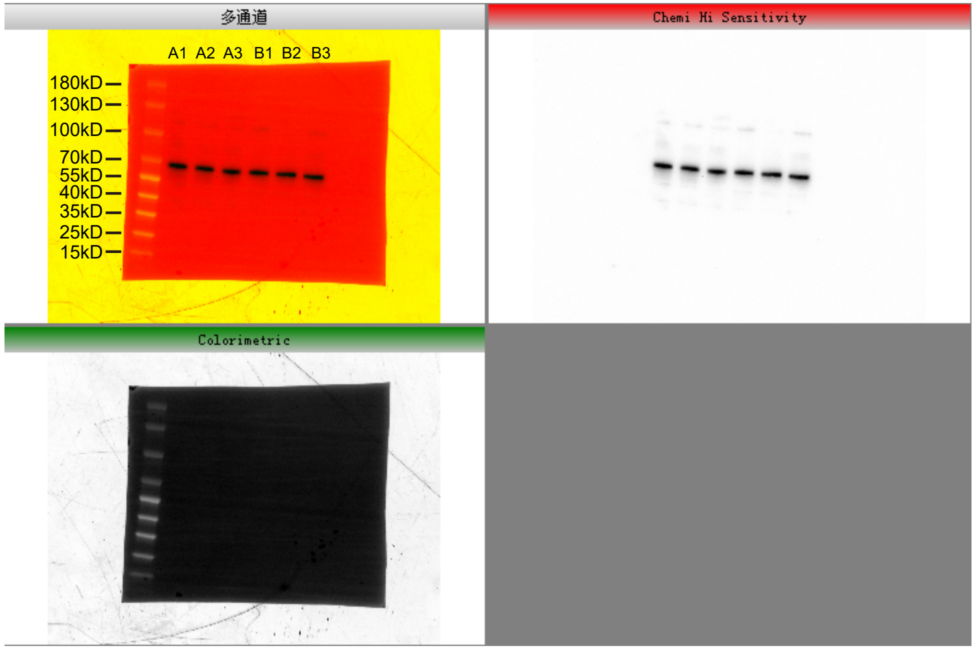

Beta Actin Monoclonal antibody (Proteintech, #66009-1-Ig)

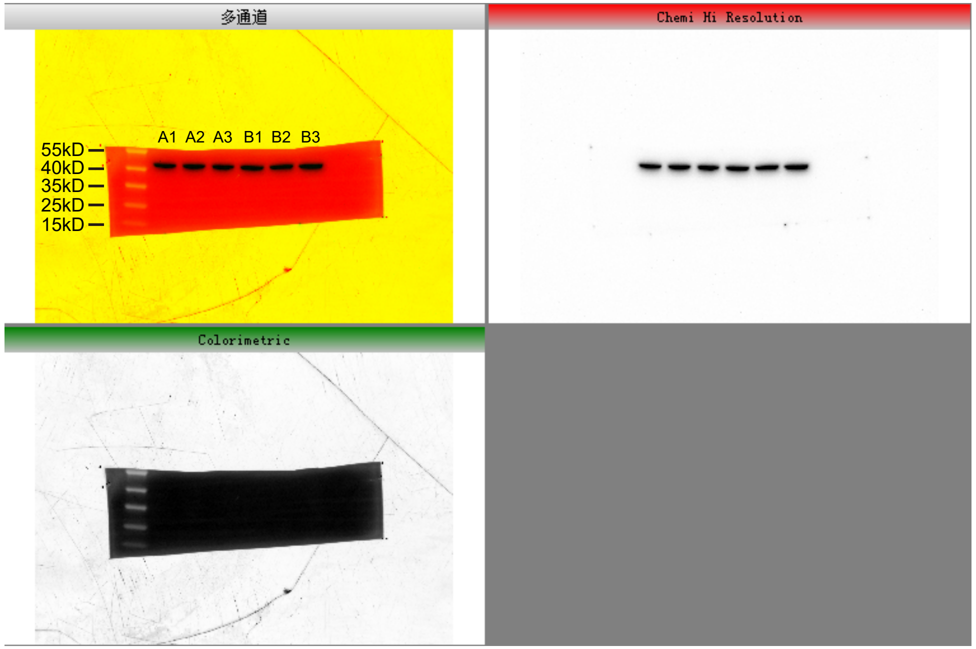

Figure 7 (A: Con; B: HS)

Phospho-ULK1 (Ser556) Recombinant monoclonal antibody (Proteintech, #80218-1-RR)

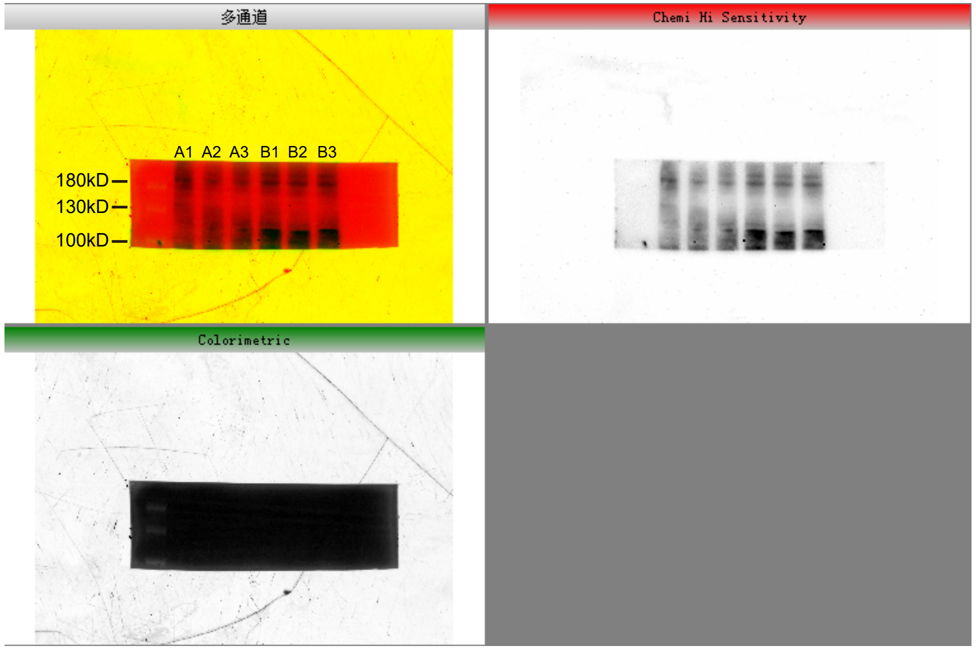

ULK1 Polyclonal antibody (Proteintech, #20986-1-AP)

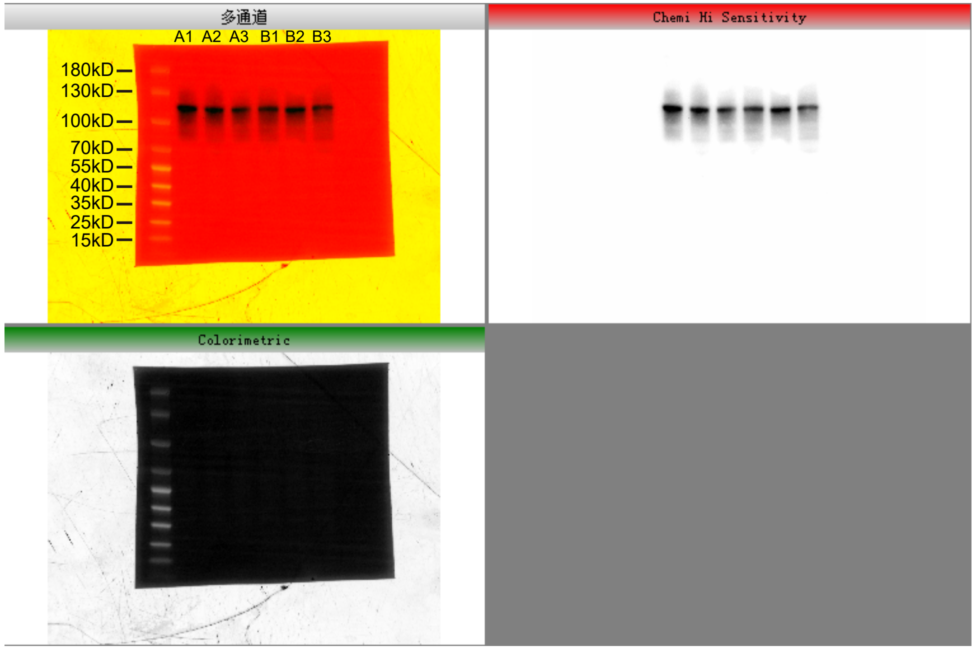

Beta Actin Monoclonal antibody (Proteintech, #66009-1-Ig)

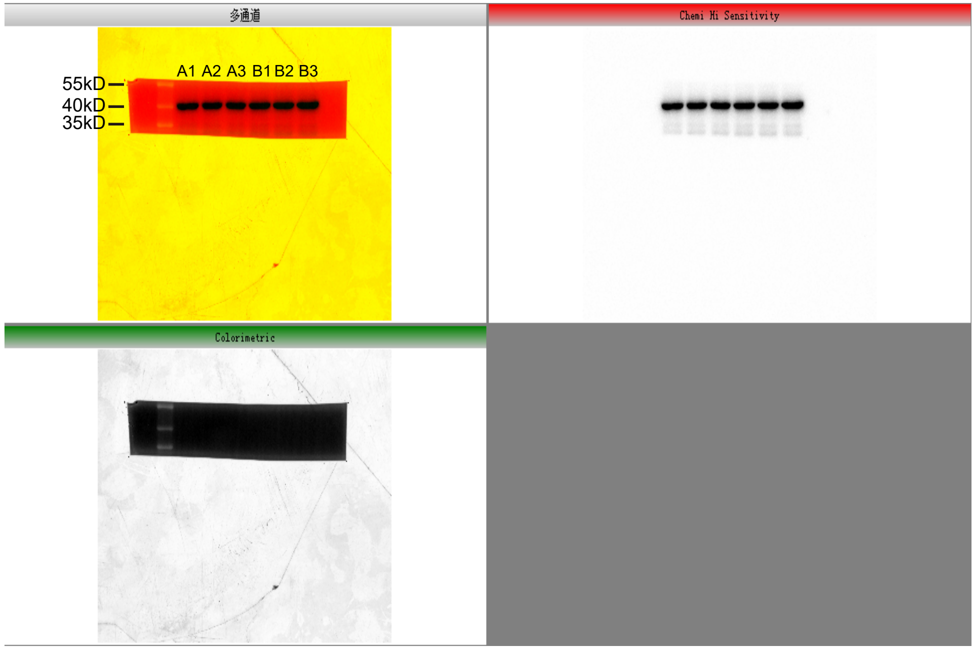

Figure 7 (A: Con; B: HS)

NCOA4 Recombinant monoclonal antibody (Proteintech, #83394-4-RR)

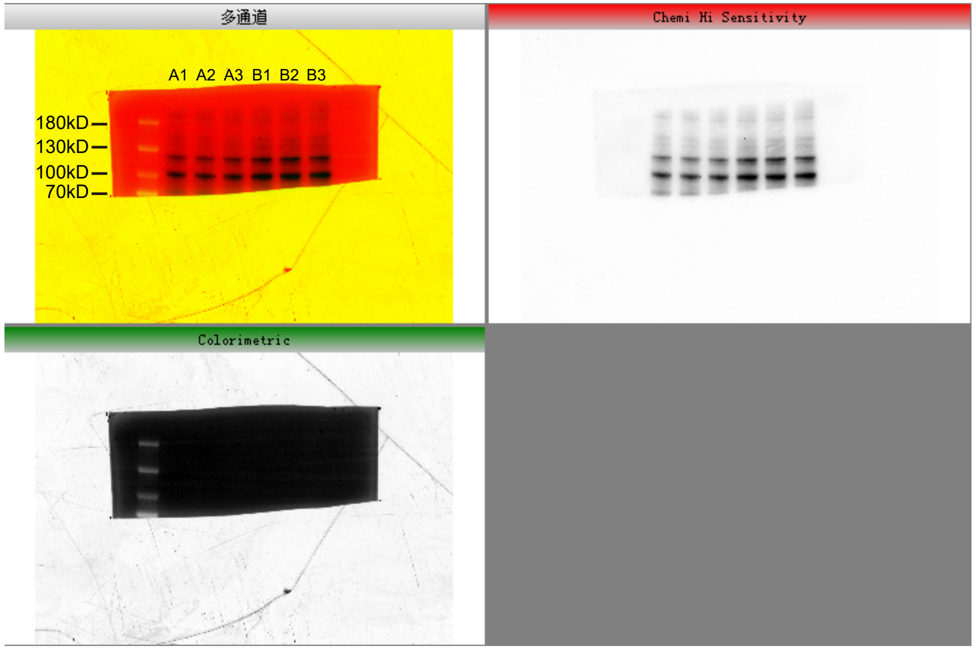

Beta Actin Monoclonal antibody (Proteintech, #66009-1-Ig)

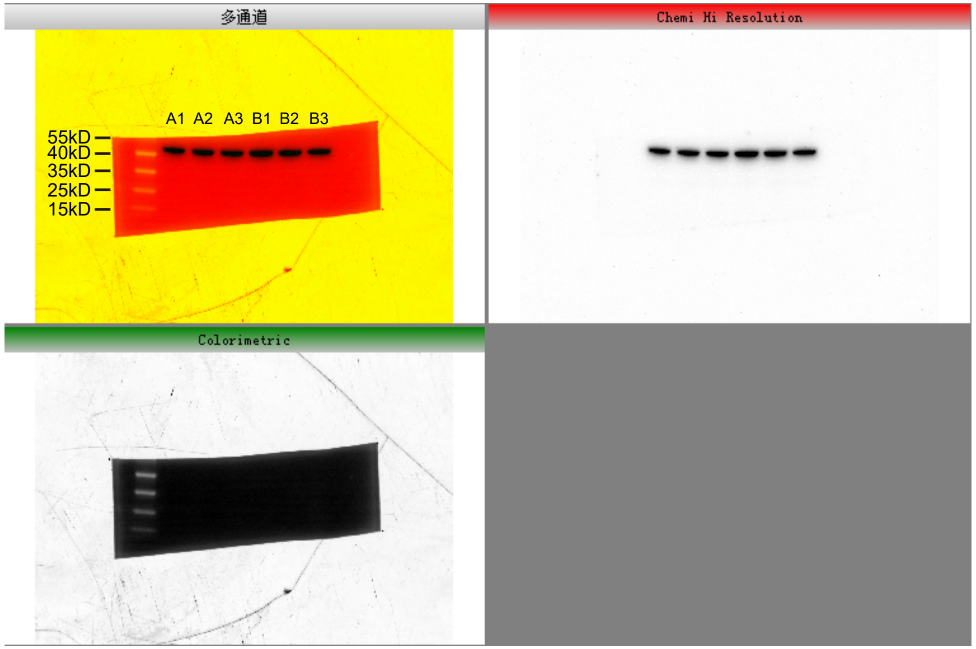

Figure 7 (A: Con; B: HS)

TFR1 Polyclonal antibody (Proteintech, #10084-2-AP)

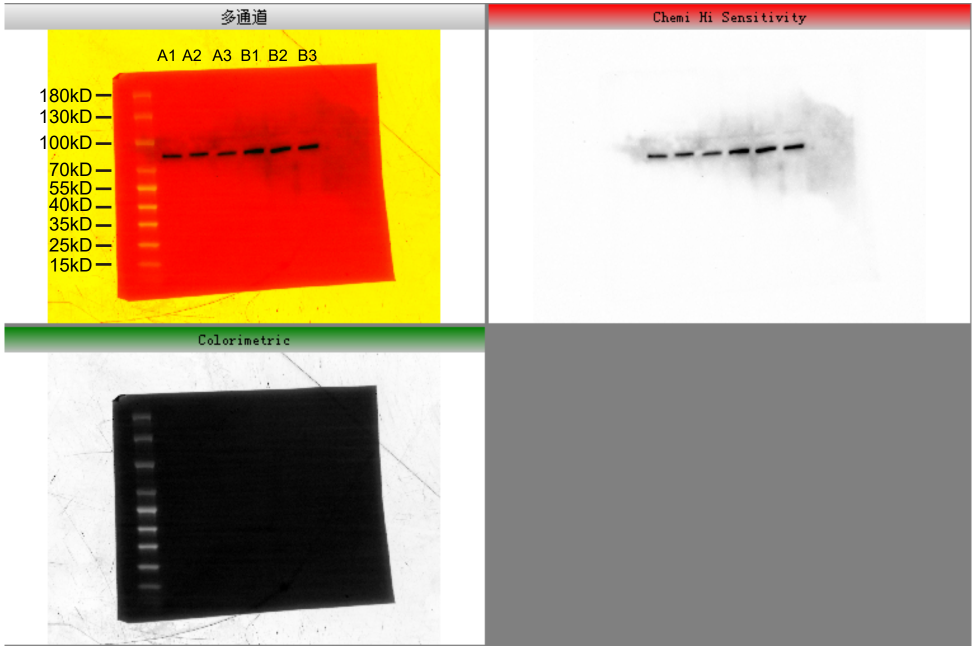

Beta Actin Monoclonal antibody (Proteintech, #66009-1-Ig)

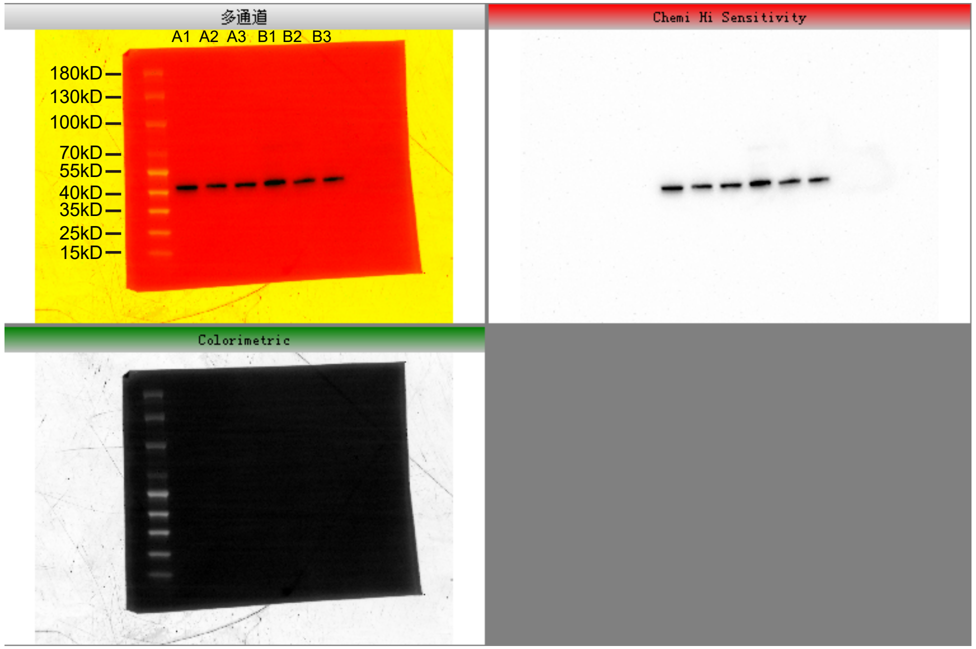

Figure 7 (A: Con; B: HS)

ACSL4 Polyclonal antibody (Proteintech, #22401-1-AP)

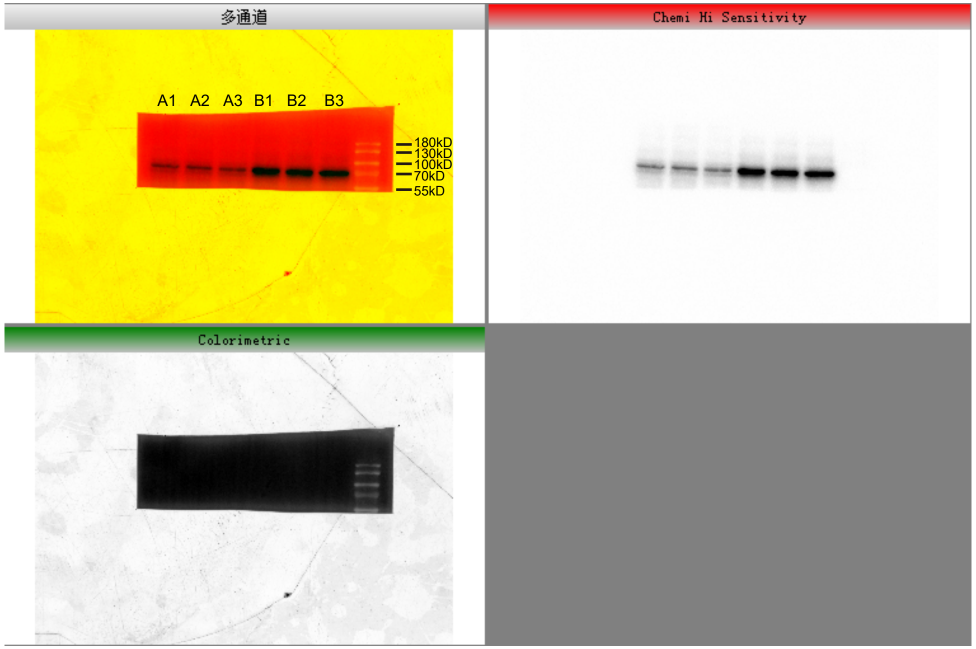

Ferritin heavy chain Recombinant monoclonal antibody (Proteintech, #83428-1-RR)

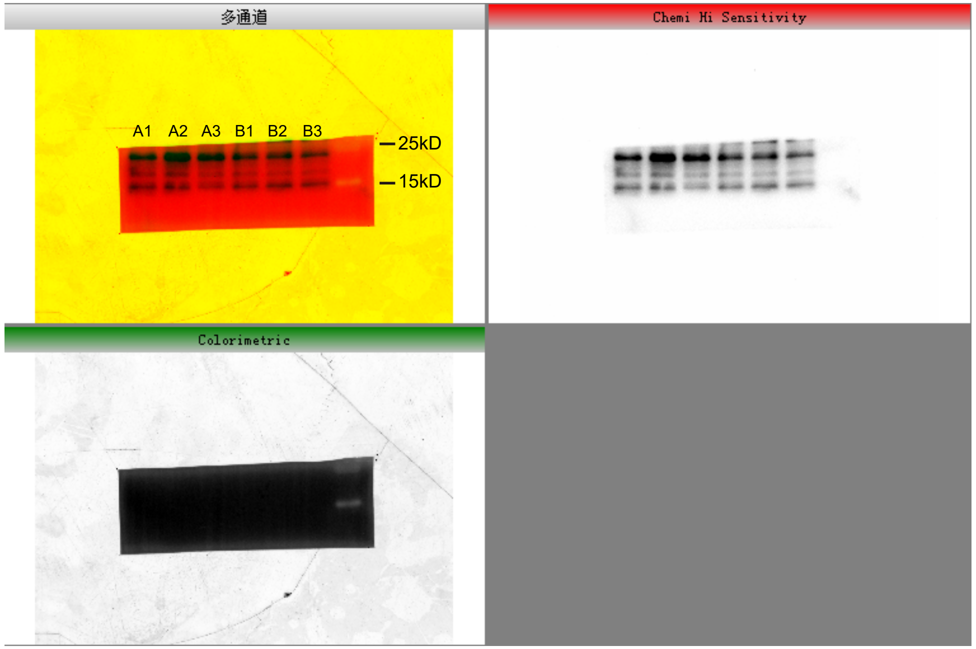

Beta Actin Monoclonal antibody (Proteintech, #66009-1-Ig)

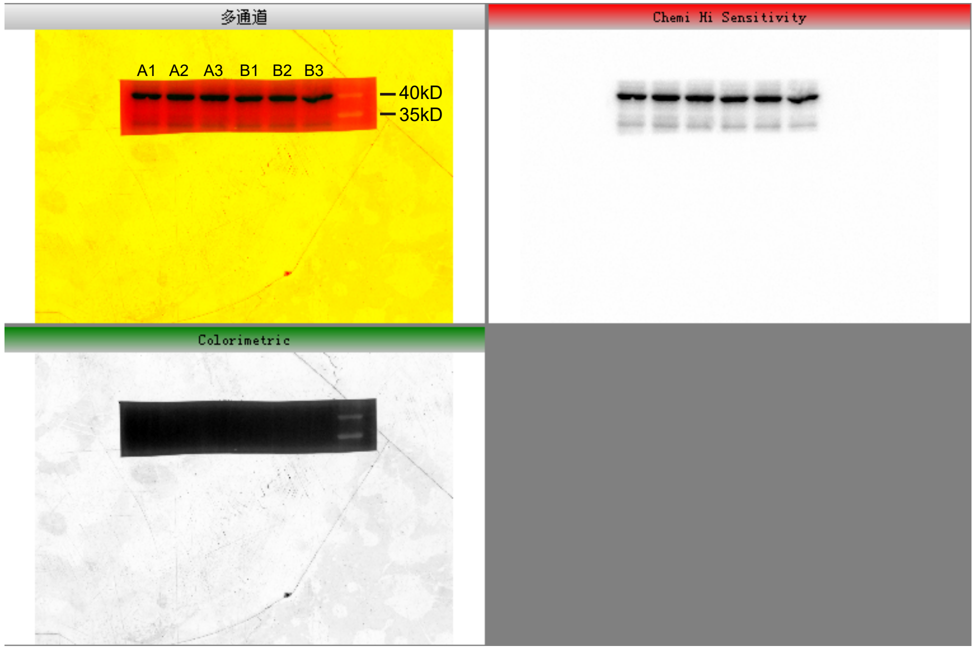

Figure 7 (A: Con; B: HS)

SLC7A11 Polyclonal antibody (Proteintech, #26864-1-AP)

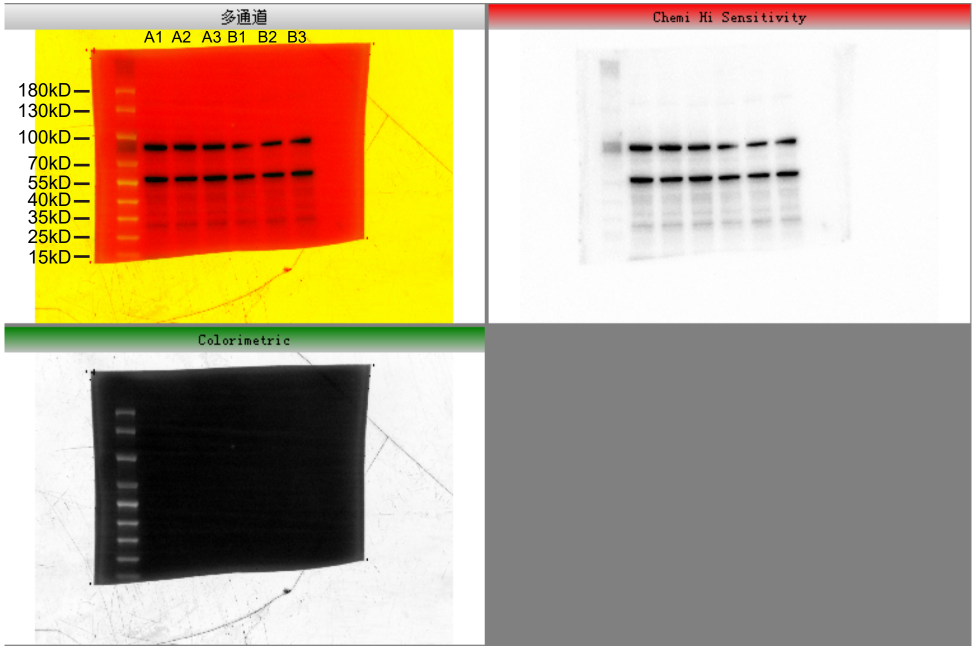

Beta Actin Monoclonal antibody (Proteintech, #66009-1-Ig)

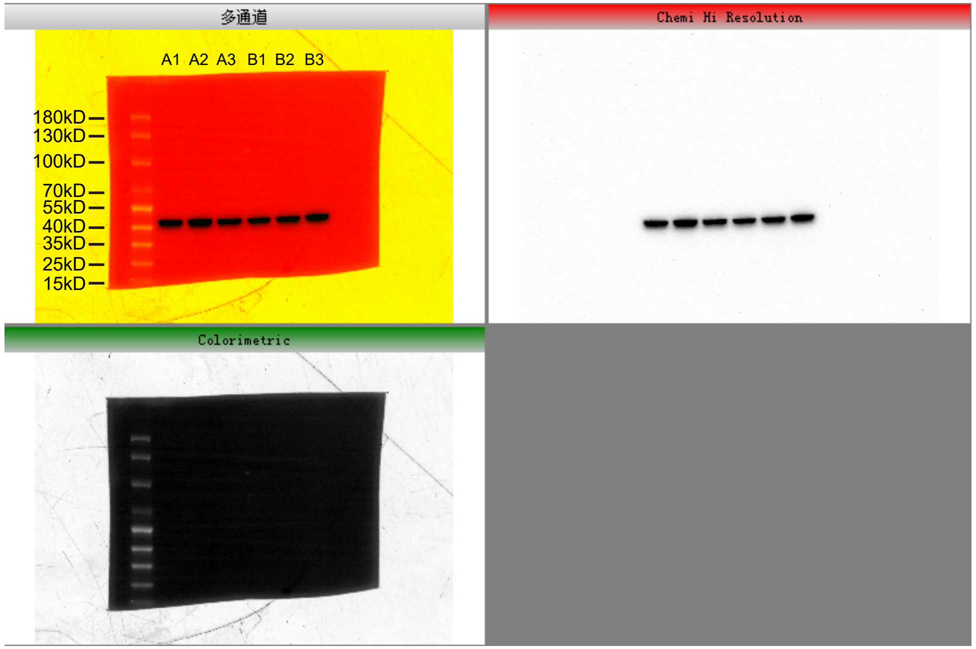

Figure 7 (A: Con; B: HS)

GPX4 Polyclonal antibody (Proteintech, #30388-1-AP)

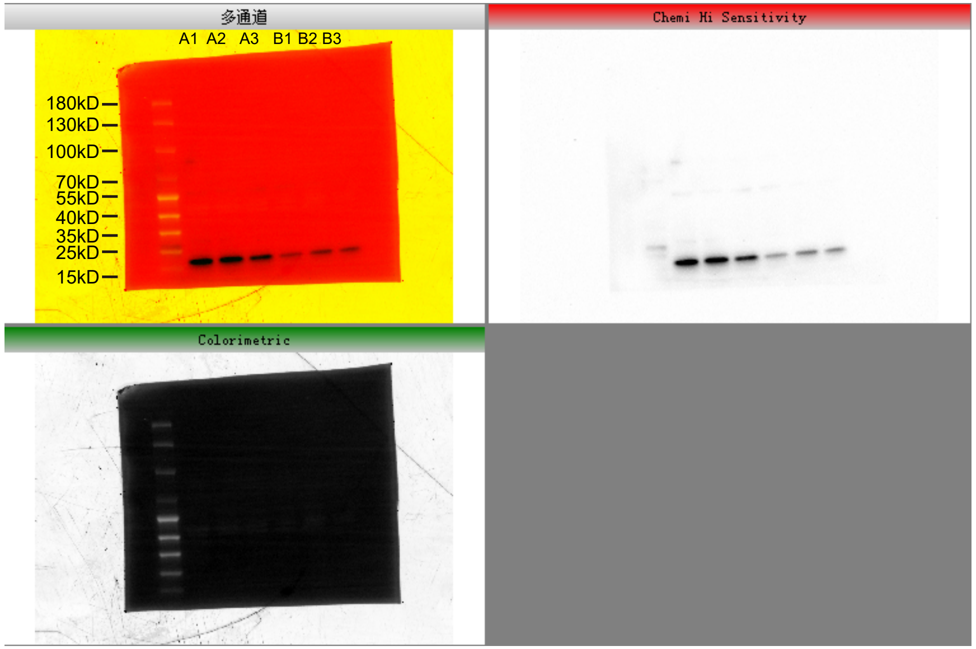

Beta Actin Monoclonal antibody (Proteintech, #66009-1-Ig)

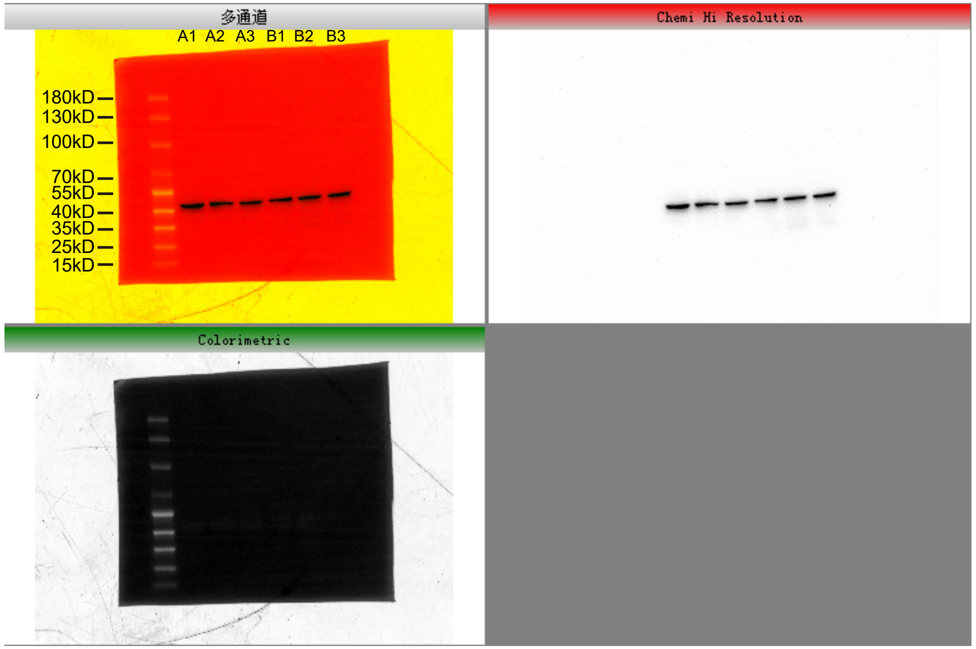

Figure 9 (A: 0 h; B: 1 h; C: 2 h; D: 3 h)

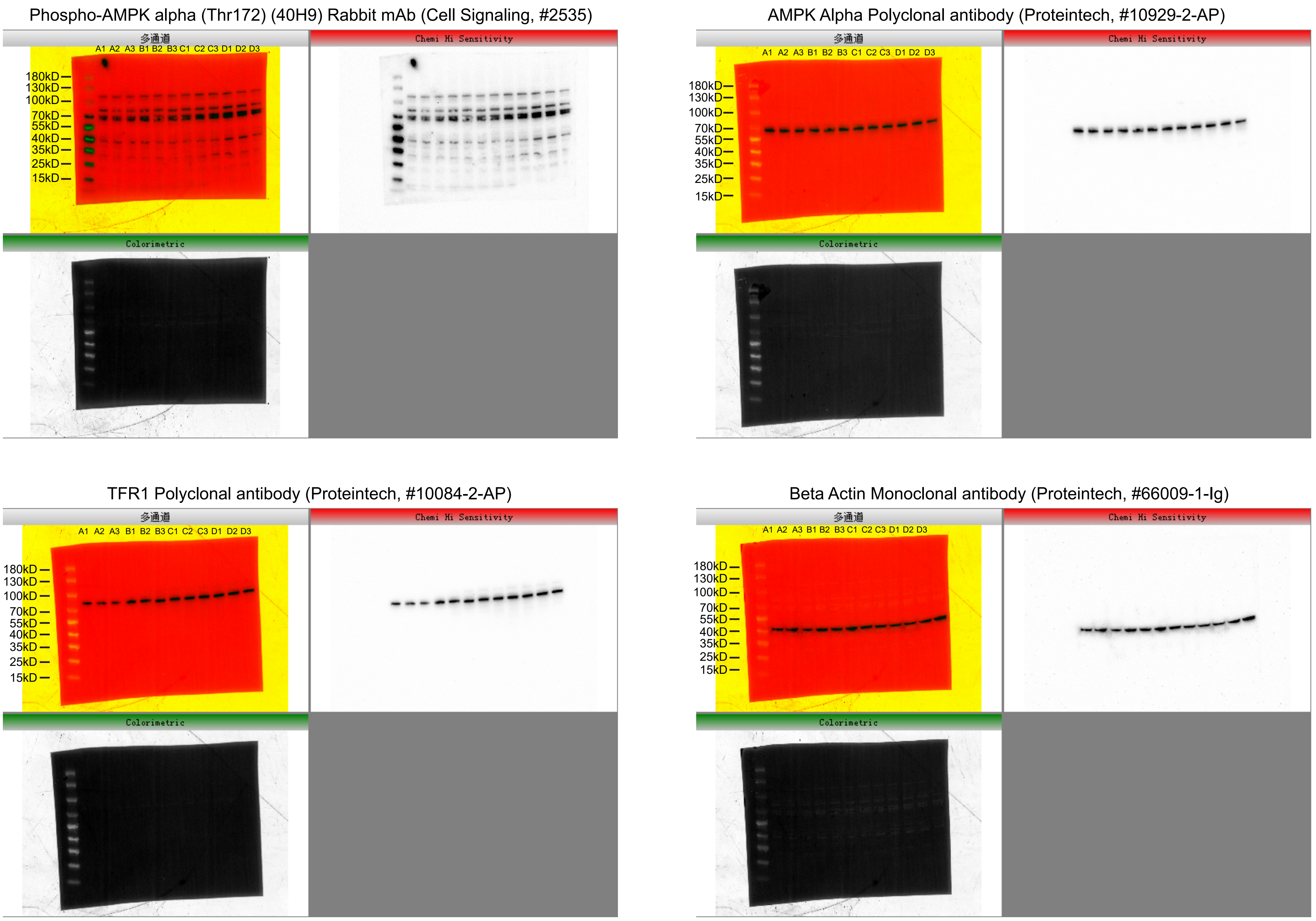

Figure 9 (A: 0 h; B: 1 h; C: 2 h; D: 3 h)

Phospho-ULK1 (Ser556) Recombinant monoclonal antibody (Proteintech, #80218-1-RR)

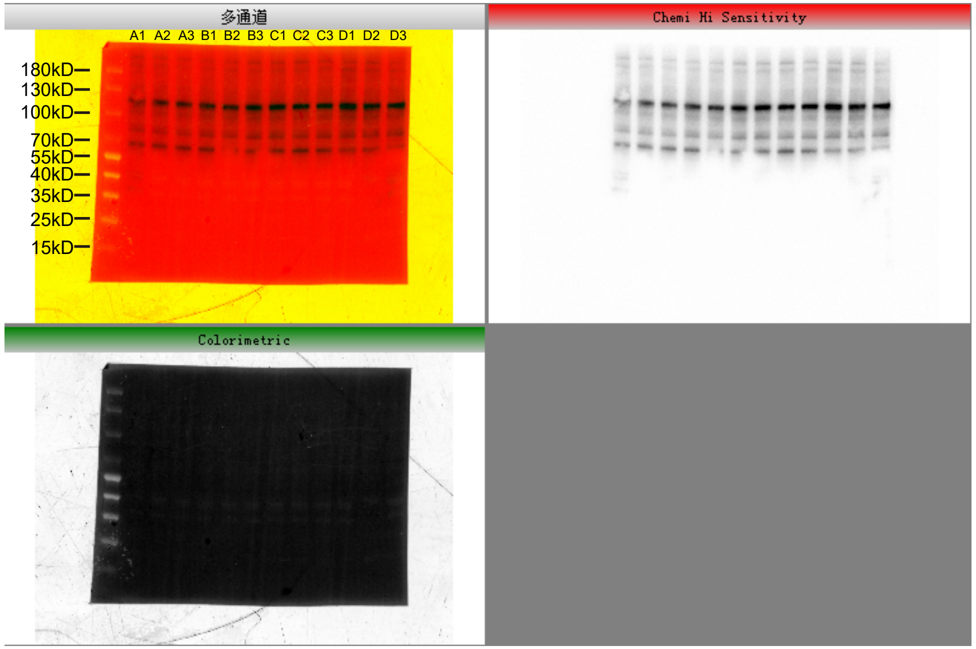

NCOA4 Recombinant monoclonal antibody (Proteintech, #83394-4-RR)

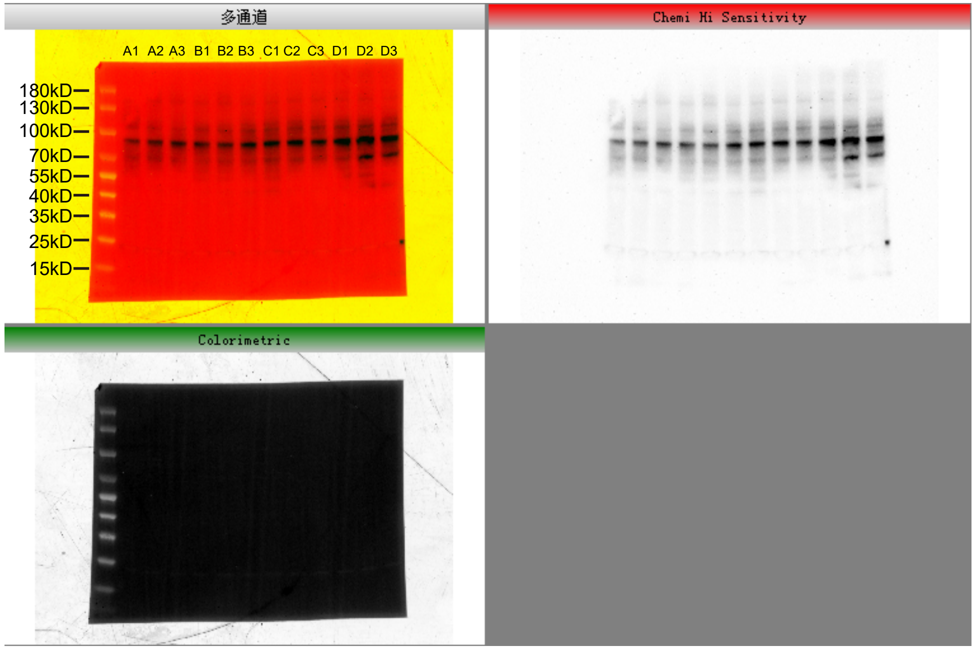

Beta Actin Monoclonal antibody (Proteintech, #66009-1-Ig)

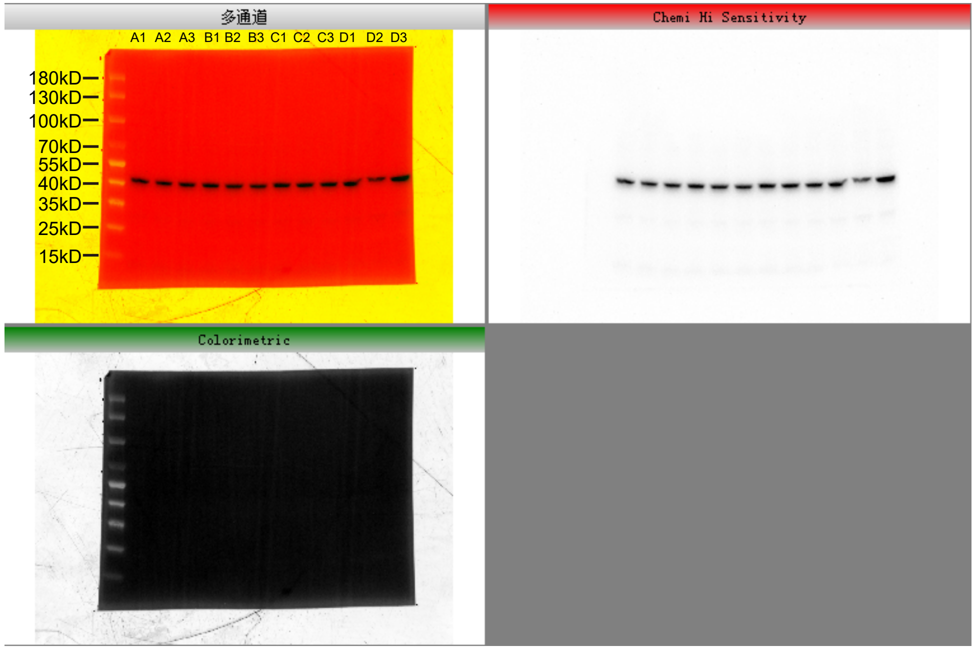

Figure 9 (A: 0 h; B: 1 h; C: 2 h; D: 3 h)

ACSL4 Polyclonal antibody (Proteintech, #22401-1-AP)

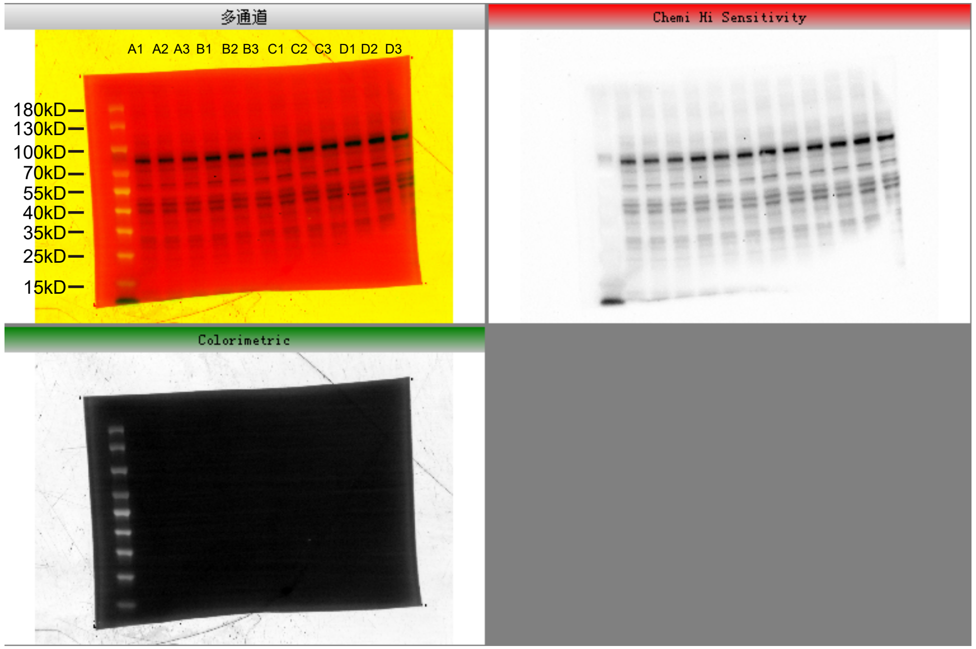

ULK1 Polyclonal antibody (Proteintech, #20986-1-AP)

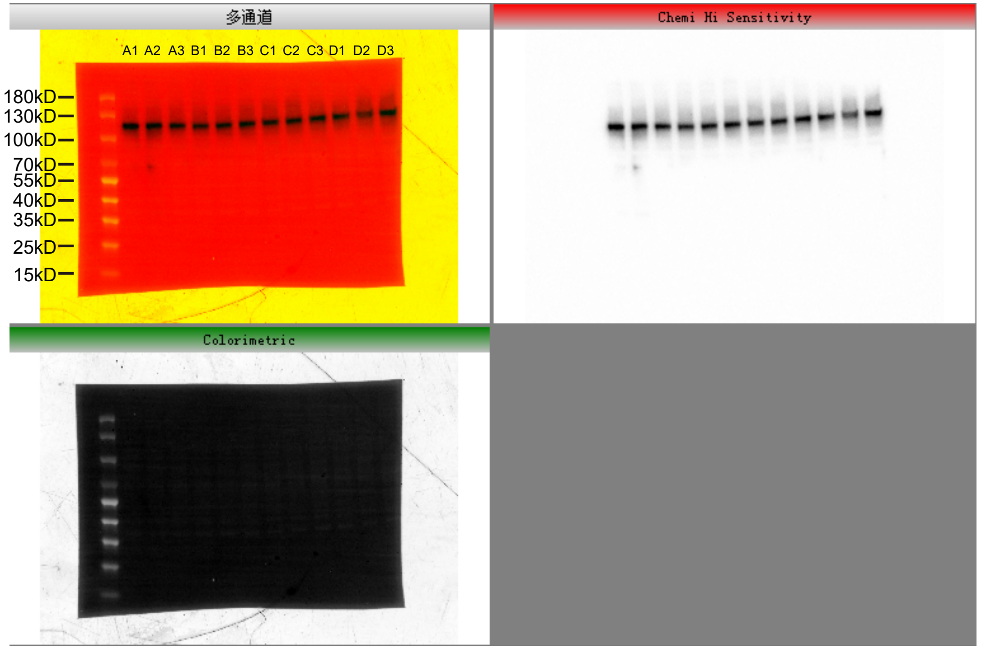

Beta Actin Monoclonal antibody (Proteintech, #66009-1-Ig)

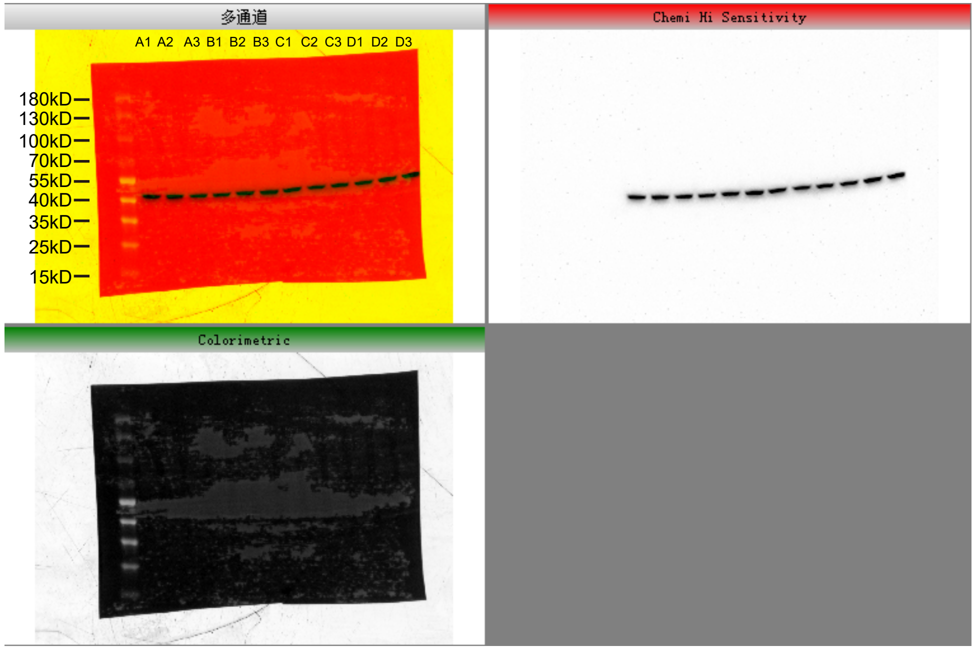

Figure 9 (A: 0 h; B: 1 h; C: 2 h; D: 3 h)

Ferritin heavy chain Recombinant monoclonal antibody (Proteintech, #83428-1-RR)

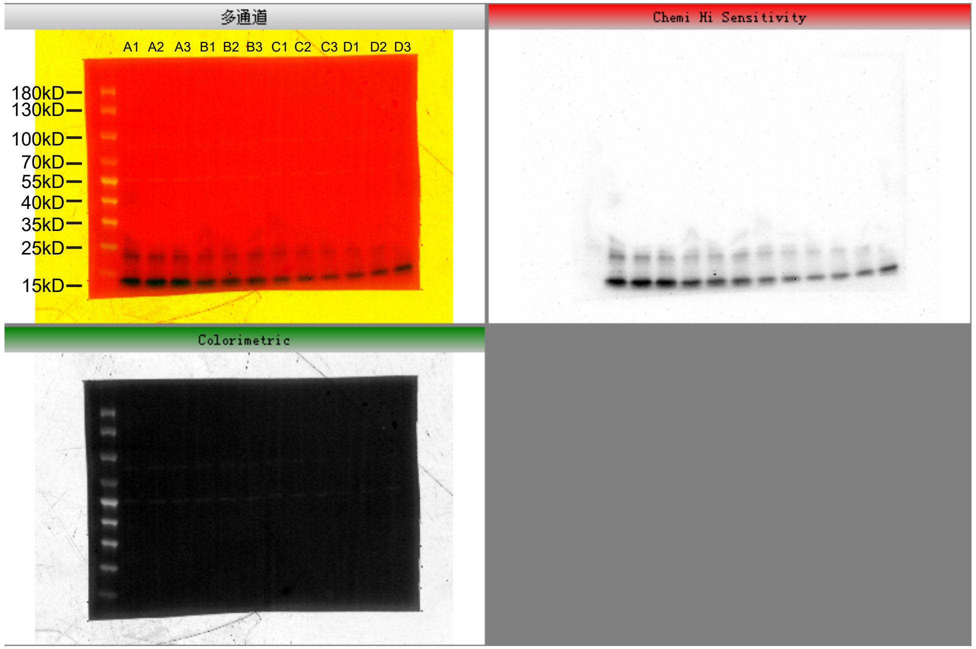

SLC7A11 Polyclonal antibody (Proteintech, #26864-1-AP)

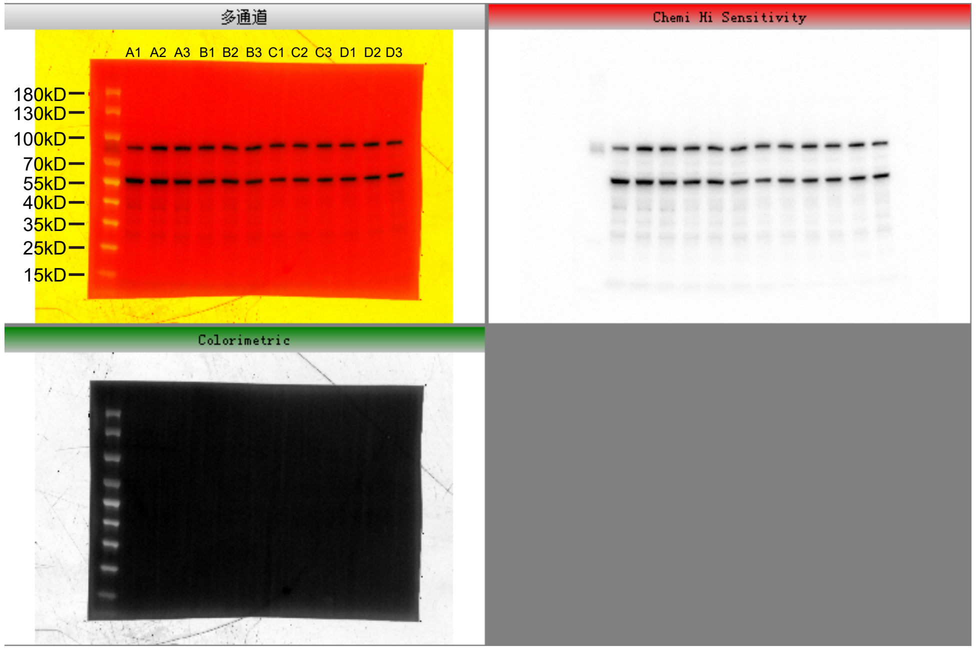

Beta Actin Monoclonal antibody (Proteintech, #66009-1-Ig)

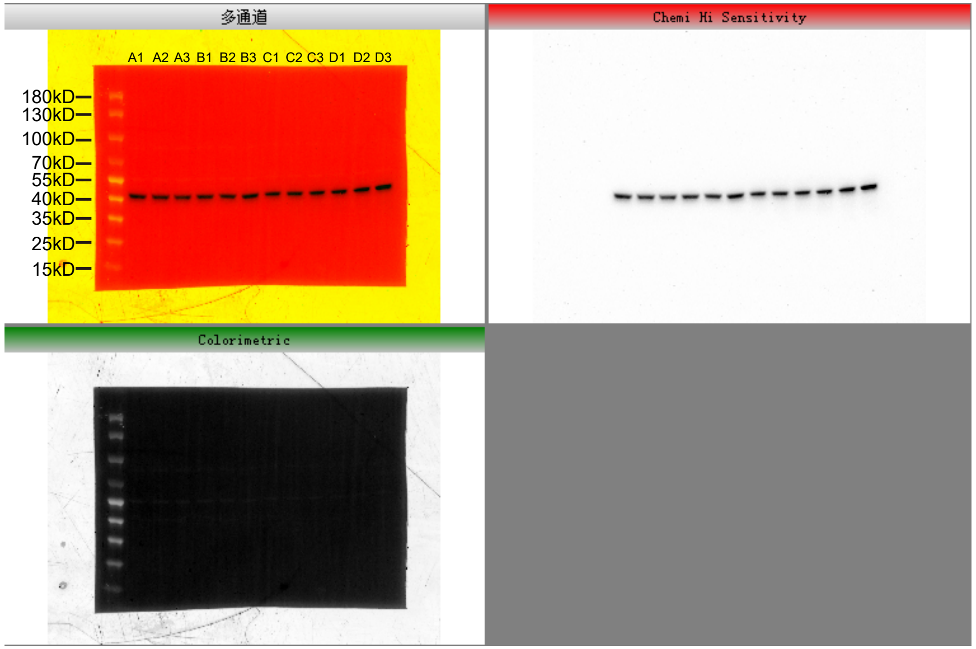

Figure 9 (A: 0 h; B: 1 h; C: 2 h; D: 3 h)

GPX4 Polyclonal antibody (Proteintech, #30388-1-AP)

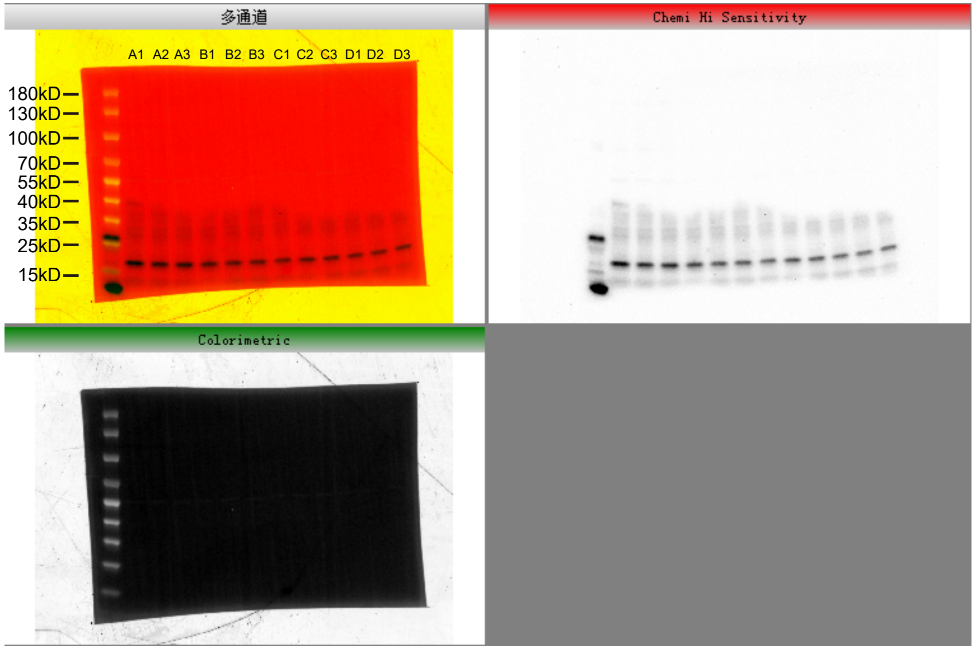

Beta Actin Monoclonal antibody (Proteintech, #66009-1-Ig)

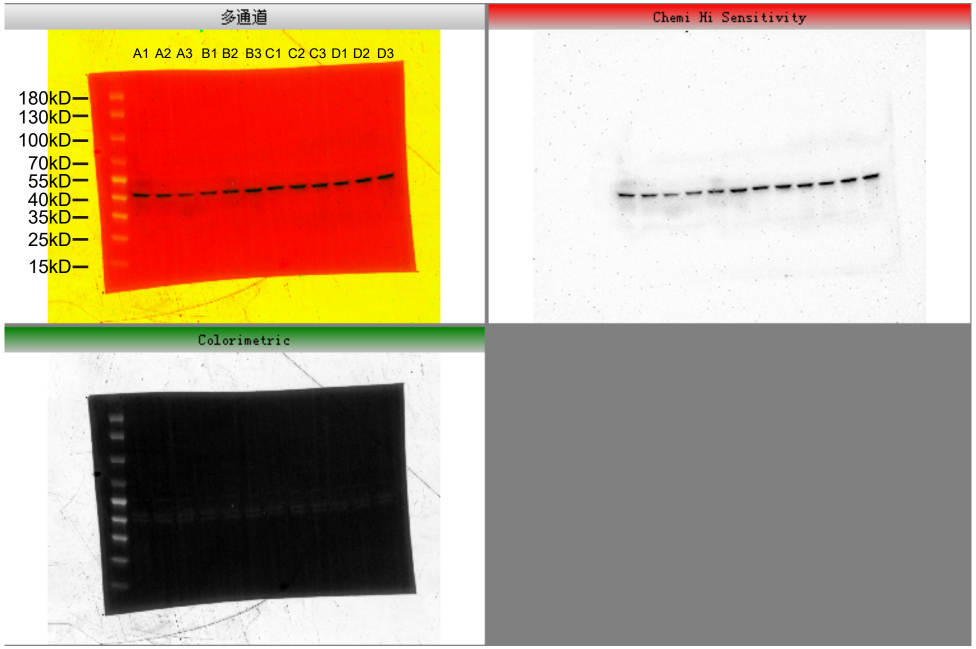

Supplement: Supplementary file 2 [file mmc2.pdf]
